# Clustering and trajectories of key noncommunicable disease risk factors in Norway: the NCDNOR project

**DOI:** 10.1038/s41598-023-41660-x

**Published:** 2023-09-02

**Authors:** Knut Eirik Dalene, Simon Lergenmuller, Erik R. Sund, Laila A. Hopstock, Trude Eid Robsahm, Yngvar Nilssen, Wenche Nystad, Inger Kristin Larsen, Inger Ariansen

**Affiliations:** 1 Department of Chronic Diseases, Norwegian Institute of Public Health, PO Box 222, 0213 Oslo, Skøyen Norway; 2 Department of Registration, Cancer Registry of Norway, PO Box 5313, 0304 Oslo, Majorstuen Norway; 3 Department of Public Health and Nursing, Norwegian University of Science and Technology, Oslo, Norway; 4 Levanger Hospital, Nord-Trøndelag Hospital Trust, Levanger, Norway; 5 Department of Health and Care Sciences, UiT The Arctic University of Norway, Oslo, Norway; 6 Department of Research, Cancer Registry of Norway, Oslo, Norway

**Keywords:** Biomarkers, Risk factors, Cancer, Type 2 diabetes, Diabetes, Metabolic syndrome, Obesity, Cardiovascular diseases, Addiction, Anxiety, Depression, Chronic obstructive pulmonary disease, Osteoporosis, Biomarkers, Epidemiology

## Abstract

Noncommunicable diseases (NCDs) are a leading cause of premature death globally and have common preventable risk factors. In Norway, the NCDNOR-project aims at establishing new knowledge in the prevention of NCDs by combining information from national registries with data from population-based health studies. In the present study, we aimed to harmonize data on key NCD risk factors from the health studies, describe clustering of risk factors using intersection diagrams and latent class analysis, and identify long-term risk factor trajectories using latent class mixed models. The harmonized study sample consisted of 808,732 individuals (1,197,158 participations). Two-thirds were exposed to ≥ 1 NCD risk factor (daily smoking, physical inactivity, obesity, hypertension, hypercholesterolaemia or hypertriglyceridaemia). In individuals exposed to ≥ 2 risk factors (24%), we identified five distinct clusters, all characterized by fewer years of education and lower income compared to individuals exposed to < 2 risk factors. We identified distinct long-term trajectories of smoking intensity, leisure-time physical activity, body mass index, blood pressure, and blood lipids. Individuals in the trajectories tended to differ across sex, education, and body mass index. This provides important insights into the mechanisms by which NCD risk factors can occur and may help the development of interventions aimed at preventing NCDs.

## Introduction

Noncommunicable diseases (NCDs) accounted for ~ 41 million (74%) of all deaths in 2019, and > 17 million died from NCDs before age 70.^1^ NCDs represent an individual burden,^2,3^ and substantial global economic costs.^4^ In response, the World Health Organization (WHO) and United Nations aim to reduce premature NCD mortality by 33% within 2030.^5,6^ Key modifiable NCD risk factors include the behavioural factors tobacco use, physical inactivity, an unhealthy diet, and harmful use of alcohol, and the biological factors obesity, hypertension, hyperlipidaemia and hyperglycaemia.^7^ While studies have investigated behavioural and biological risk factors separately,^8–11^ few have explored the clustering of these factors combined.^12–14^ Previous research has identified risk factor patterns in different populations, using various methods,^8,9,15,16^ but large population-based studies in adults from westernized countries using appropriate model-based techniques are lacking. Furthermore, studies have indicated that risk factor patterns may vary over time,^17–21^ yet to our knowledge, few studies investigated trajectories of three or more risk factors in prospectively collected data.^18,19^ As longevity increases, it is likely that NCD prevalence will increase, but this may not affect all population groups equally. Hence, to counteract further widening of social inequality in health,^22^ we need to identify vulnerable groups of individuals who cluster with respect to NCD risk factors.

“A life-course approach to prevent noncommunicable diseases in an ageing population – NCDNOR”, is a research project aiming at establishing new knowledge in the prevention of NCDs by combing NCD endpoints across somatic disciplines, examining effects of socioeconomic circumstances, health behaviours, biological markers, and mental health throughout the life-course. The project utilizes unique data from administrative and health registries covering the entire Norwegian population and includes information on both behavioural and biological NCD risk factors from Norwegian population-based health studies covering ~ 800,000 individuals nationwide and ~ 1.2 million person-observations. In the present study, we aim to (i) describe the health studies included in NCDNOR and the harmonization of data on key NCD risk factors to create one study sample, (ii) describe the clustering of key NCD risk factors, and (iii), for individuals with repeated measurements, identify trajectories of key NCD risk factors.

## Methods

### Study sample

**Figure 1:**
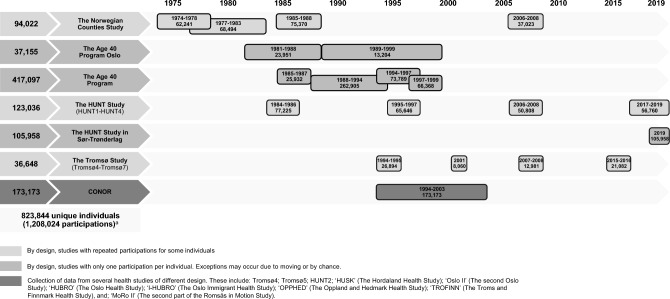
Timeline for the health studies included in this study. Abbreviations: HUNT, The Trøndelag Health Study; CONOR, Cohort of Norway. ^a^CONOR includes data from HUNT2 and Tromsø4-Tromsø5. These participations were only counted once.

The NCDNOR project includes health study data from women and men who participated in at least one of the following studies: the Norwegian Counties Study^23,24^; the Age 40 Program Oslo^25^; the Age 40 Program^26^; the Trøndelag Health Studies (HUNT1–HUNT4/HUNT4ST)^27^; the Tromsø Study (Tromsø4–Tromsø7)^28^; and/or; the Cohort of Norway (CONOR) studies^29^ (Fig. 1). The different studies have been described elsewhere,^23–29^ and thus only briefly described in the following. More details are given in Supplementary Table S1.

**Figure 2:**
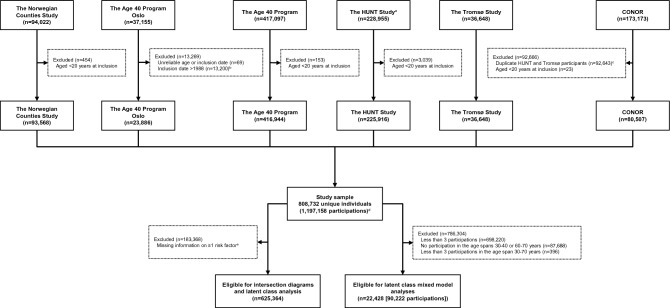
Selection of individuals from the different health studies into the study samples. Abbreviations: HUNT, The Trøndelag Health Study; CONOR, Cohort of Norway. ^a^Including HUNT Sør-Trøndelag, ^b^The quality of the data collected after 1988 was reported to be poor. ^c^CONOR includes data from HUNT2 and Tromsø4-Tromsø5. We excluded those from CONOR. ^d^808,732 does not correspond to the sum of the numbers at the bottom of each health study, as individuals may have participated in several different studies. In addition, 181 individuals participated to several health studies < 1 year apart. These participations are not included in 1,197,158. ^e^Missing information consists of 77.6% non-measured, non-response (20.7%) and manual cleaning decisions (1.7%).

*The Norwegian Counties Study* included participants from three Norwegian counties, with three waves of data collection 1974–1988 (Fig. 1). For these first three waves (participation 84–88%),^30^ all residents aged 35–49 years and random samples of residents aged 20–34 were invited. The second and third wave also invited previous participants. In 2006–2008, a fourth wave was conducted among a sample of previous participants (participation 59%).^24^ We included 93,568 individuals from the Norwegian Counties Study (Fig. 2), of which > 50,000 individuals participated at least three times.

*The Age 40 Program Oslo* was a cardiovascular disease (CVD) risk factor screening program conducted by the Oslo municipality health council in collaboration with the National Health Screening Service in Norway.^31^ All residents who turned 40 years between 1981 and 1988 were invited the year they turned 40 (participation 55%).^25^ A restructured program continued until 1999. Due to poor quality of the data, we did not include data collected after 1988.^25^ We included 23,886 individuals from the Age 40 Program Oslo (Fig. 2).

*The Age 40 Program* was similar to the Age 40 Program Oslo and conducted by the National Health Screening Service in the other Norwegian counties in 1985–1999. All residents aged 40–42 years were invited.^26^ In addition, some counties invited residents aged < 40, 43–44 and 65–67 years.^32^ Some individuals moved between counties, resulting in ~ 10,000 individuals participating several times. The overall participation was ~ 69% (81% in 1985, 52% in 1999).^26^ We included 416,944 individuals from the Age 40 Program (Fig. 2).

*The HUNT Study* has been conducted in four waves (1984–2019) (Fig. 1).^27^ All residents ≥ 20 years of age in the former Nord-Trøndelag county were invited (participation 89% (HUNT1), 54% (HUNT4)). In 2019, HUNT also invited all residents aged ≥ 18 years in the former Sør-Trøndelag county (HUNT4ST, participation 43%). In total, ~ 250,000 individuals have participated, yielding ~ 380,000 person-observations. We included 225,916 individuals from the HUNT Study (Fig. 2).

*The Tromsø Study* has been conducted seven times (1974–2016).^28^ NCDNOR includes individuals from Tromsø4–Tromsø7 (Fig. 1). In Tromsø4 and Tromsø7 all residents of Tromsø municipality aged ≥ 25 and ≥ 40 years were invited, respectively. In Tromsø5 and Tromsø6, previous participants and age-group specific random samples were invited (participation 65–79%).^33^ We included 36,648 individuals from Tromsø4–Tromsø7 (Fig. 2), of which ~ 20,000 individuals participated at least two times.

*CONOR* results from a collaboration between epidemiological research centres in Norway, and includes harmonized data from 10 regional health studies (including HUNT2, Tromsø4 and Tromsø5, Fig. 1).^29^ Some studies invited everyone above a specific age, others invited selected age groups.^34^ After excluding the duplicate HUNT and Tromsø participants, we included 80,507 individuals from the remaining seven CONOR studies (participation 30–66%) (Fig. 2).

NCDNOR also includes health study data from the Norwegian Youth Health Studies,^32^ and the Young-HUNT studies^27^ not included in the present study.

### Harmonization of data on NCD risk factors

The protocols of the health studies included in NCDNOR were similar,^31^ facilitating harmonization and pooling of data. All studies included questionnaires covering behavioural factors. Except for HUNT4ST and the 4^th^ Norwegian Counties Study, studies included anthropometric and biomarker measurements conducted by trained personnel. A full description of the data can be found elsewhere.^33,35,36^ Based on data-availability, and the global strategies for the prevention of NCDs,^7^ we consider the following key NCD risk factors: *daily smoking*, *physical inactivity*, *harmful use of alcohol* (only available for a subsample), *obesity*, *hypertension*, *hypercholesterolaemia* and *hypertriglyceridaemia*. A brief description of data harmonization and risk factor definitions follows (additional details in Supplementary Tables S2–S4).

#### Behavioural risk factors

Questions about smoking varied between studies, but all questionnaires allowed categorization of participants into smoking status (never, former, or current daily smokers [risk factor *daily smoking*]), and calculation of *smoking intensity* (cigarettes/day [currently or previously]) and smoking duration (years) (Supplementary Tables S2a & S2b).

Leisure-time physical activity (*LTPA*) was assessed either by the Saltin-Grimby Physical Activity Level Scale (SGPALS), ‘the CONOR instrument’,^37^ ‘the HUNT instrument’,^38^ or a combination (Supplementary Tables S3a-d).^39^ These were harmonized into the SGPALS categories (Supplementary Tables S3e-f): (1) Reading, TV-watching, or other sedentary activities (risk factor *physical inactivity*), (2) Riding a bicycle or walking (including to/from work) or doing other activities of light-moderate intensity ≥ 4 h/week, (3) Heavy gardening, sports, exercise ≥ 4 h/week, and  (4) Regular hard physical exercise, participation in competitive sports, etc. several times/week.

Information on frequency of alcohol consumption, alcohol units usually consumed over 14 days, frequency of heavy episodic drinking, and alcohol problems were available for a subset of studies (Supplementary Table S4). We define *harmful use of alcohol* as > 5 units of beer, wine or spirits on at least one occasion in the past 30 days (corresponding to ≥ 59 g of pure alcohol) or self-reported alcohol problems.^40,41^ We also calculated *mean alcohol consumption* (grams of pure alcohol/14 days) by multiplying units of beer (0.33L), wine (0.15L) and spirits (0.04L) by 11.8, 14.0 and 12.6, respectively^41^.

#### Biological risk factors

With two exceptions, height and weight were measured by trained staff (self-reported in HUNT4ST and the 4^th^ Norwegian Counties Study). We removed extreme values (< 120/ > 220 cm, < 30/ > 260 kg) and calculated *BMI* (kg/m^2^). *BMI* ≥ 30 defined *obesity* (values < 10/ > 80 were set to missing). For the 4^th^ Norwegian Counties Study, only weight was self-reported, and BMI calculated using height measured in the 1^st^, 2^nd^ or 3^rd^ Norwegian Counties Study^24^.

In the 1^st^ and 2^nd^ Norwegian Counties Study, HUNT1, and the Age 40 Program Oslo, blood pressure was measured twice using calibrated mercury sphygmomanometers (≥ 1-min interval) after ≥ 4 min seated rest.^42^ Thereafter, three automatic oscillometric blood pressure measurements were taken (1-min intervals) after ≥ 2 min seated rest. We harmonized measurements using the second sphygmomanometer measurement and the rounded arithmetic mean of oscillometric measurements two and three.^43,44^ If only two oscillometric measurements were available, we used the last measurement.^43^ We excluded extreme blood pressure values (diastolic < 30/ > 160 mmHg, systolic < 60 mmHg). In the present study, we defined* hypertension* as a *systolic blood pressure* ≥ 160 mmHg and/or *diastolic blood pressure* ≥ 100 mmHg (Grade 2 or 3 hypertension),^44^ as outlined in the national guidelines for the prevention of cardiovascular disease when considering blood pressure as an individual risk factor^45^.

Serum *total cholesterol*- and *triglycerides* were analysed from non-fasting, venous blood samples using the non-enzymatic Liebermann-Burchard method (Technicon Autoanalyzer) until 1979.^46^ Thereafter, analyses were conducted using enzymatic methods. Results obtained before 1979 were recalculated using a correction factor (enzymatic method = 0.92 × non-enzymatic method + 0.03).^46^ Total cholesterol < 2 mmol/L or > 20 mmol/L and triglycerides < 0.01 mmol/L or > 30 mmol/L were set to missing.^47,48^ We then defined the risk factors *hypercholesterolaemia* (≥ 7 mmol/L) and *hypertriglyceridaemia* (≥ 4 mmol/L), which were guided by national guidelines for the prevention of cardiovascular disease and the 2019 European Society of Cardiology and European Atherosclerosis Society Guidelines for the management of dyslipidaemias^45,48^.

#### Other variables

The health study data included in NCDNOR can be linked to national mandatory health and administrative registries that cover the entire population.^49^ We used data from Statistics Norway on geography, education and income.^50^ We categorized highest attained educational into levels corresponding to the current Norwegian standard^51^: 1) Primary or lower secondary school (Primary [≤ 10 years of education]); 2) Upper secondary education (Secondary [11–14 years]); 3) Higher education (Tertiary [> 14 years, typically a university/college degree]), and highest attained income using year and sex-specific quintiles.

### Statistical analyses

We ensured the correct ordering of the participations within individuals with repeated measurements by excluding participations < 1 year apart (n = 181, Fig. 2 (due to data protection risk minimisation, date uncertainties could range 45–365 days)). We defined the time of first participation as ‘study entry’.

We described the occurrence and clustering of NCD risk factors at study entry using intersection diagrams overall, by sex and study entry period (1974–1989, 1990–2004 and 2005–2019).^52^ We further described the clustering of risk factors using latent class analysis (LCA) to identify distinct patterns of risk factors among individuals with at least two such risk factors (Supplementary Methods)^53,54^.

To identify trajectories of risk factors among individuals with repeated participations, we used latent class mixed models (LCMMs) (Supplementary Methods 2).^54–56^ We used the continuous/categorical version of the NCD risk factors (smoking intensity [among smokers], LTPA, BMI, blood pressure, blood lipids). For blood pressure and blood lipids, we used multivariate LCMMs, allowing simultaneous modelling of trajectories.^56^ We estimated trajectories for those with at least three participations in the time window 30–70 years of age, whereof one participation had to occur within the age span 30–40 years (trajectory entry), and another within the age span 60–70 years (Supplementary Methods).

For LCA and LCMM, the optimal number of classes was identified by fitting models with 1–6 classes and evaluating each model in terms of: quality of the model fit (Akaike Information Criterion, Bayesian Information Criterion), classification power (entropy, posterior probability), and relevance.^57–59^ Once the optimal number of classes was identified, we allocated individuals to the class for which they had the largest posterior probability (Supplementary Methods).

Depending on the model, and the restrictions used to identify classes, we had various degrees of missing data (non-measured, non-response, or manual cleaning decisions). The proportion of missing was considerably higher for alcohol (Supplementary Table S5), we therefore provide results including alcohol as supplements only. We conducted several sensitivity analyses (Supplementary Methods).

### Ethics

Access to data in NCDNOR is based on informed consent from the participants, approval from The Regional Committees for Medical and Health Research Ethics South-East (nr 28,561/2019/1203), and The Data Privacy Impact Assessment at the Norwegian Institute of Public Health^49^. The legal basis for processing of personal data is Article 6 (1) (e) of the GDPR and the exemptions pursuant to Article 9 (2) (j) of the GDPR, with a supplementary legal basis in Sects. 8 and 9 of the Personal Data Act. All methods were performed in accordance with relevant guidelines and regulations including the Declarations of Helsinki.

## Results

Combined, the health studies sent out 1,905,277 invitations, resulting in 1,208,024 participations (63%) from 823,844 unique individuals (Fig. 1) covering all Norwegian counties (Supplementary Fig. S1). After exclusions, the final study sample consisted of 808,732 individuals (52% women) aged ≥ 20 years (born 1882–1999), who participated in at least one health study between 1974 and 2019, for a total of 1,197,158 participations (Fig. 2). Repeated measurements were available for 211,705 (26.2%) participants, with 110,512 (13.7%) participating at least three times and 52,222 (6.5%) participating at least four times (Table 1).

**Table 1:** Number of participations by health studies and overall (n = 808,732).

	Number of individuals in the health studies with at least *k* participations^a^						Total no. individuals with at least *k* participations
	The Norwegian Counties Study	The Age 40 Program Oslo	The Age 40 Program	The HUNT Study	The Tromsø Study	CONOR	
Part. 1, No^b^	93,568	23,884	416,935	225,914	36,648	80,507	808,732
Age^c^	41.0 (8.4)	40.3 (0.6)	41.4 (1.6)	45.5 (29.5)	45.2 (18.4)	45.5 (19.3)	
Part. 2, No	77,853		9,628	68,922	19,909	90	211,705
Age^c^	46.9 (10.8)		44.8 (3.3)	51.6 (22.3)	59.7 (14.7)	47.0 (14.1)	
Part. 3, No	51,620		2,197	38,949	9,647		110,512
Age^c^	51.4 (9.5)		54.7 (16.1)	60.4 (16.5)	66.5 (14.3)		
Part. 4, No	18,768		17	19,290	2,812		52,222
Age^c^	70.5 (6.1)		–	69.8 (13.2)	74.8 (9.9)		
Part. 5, No							10,502
Part. 6, No							2,280
Part. 7–9, No							915
Total No. Participations	241,809	23,884	428,777	353,075	69,016	80,597	1,197,158

*Part* participation, *No*. number, *HUNT* The Trøndelag Health Study, *CONOR* Cohort of Norway.

^a^The total number of individuals in each health study differs from the number of individuals in each study shown in Fig. 2, because participations < 1 year apart from each other (between studies) were excluded (n = 181). The total number of individuals in the final study sample remains unchanged.

^b^The number of individuals with at least one participation is also the total number of individuals in each health study. Individuals can participate in several different health studies, therefore, the sum of the numbers in the first row (n = 877,456) does not correspond to the total number of individuals with at least one participation in the final study sample (n = 808,732).

^c^Median (Inter Quartile Range [IQR])], not shown for the Age 40 Program Participation 4 due to low n (n = 17).

At study entry, median age was 41.4 (inter-quartile range [IQR]: 3.3) years, median birth year was 1951 (IQR: 11.5), and the median participation year was 1993 (IQR: 11.2) (Table 2). Cohort characteristics stratified by participation number for those that participated at least three times are shown in Supplementary Table S6. For these, the median age at study entry was 39.4 years (IQR: 11.7), and median birth year 1940 (IQR: 16.7). The median time between participations was 5.0 (IQR: 6.0) years between participation 1 and 2, and 9.4 (IQR: 6.0) years between participation 2 and 3.

**Table 2:** Participant characteristics at study entry (n = 808,732).

Characteristic	Women	Men	Overall
Participants, No. (%)	420,840 (52.0)	387,892 (48.0)	808,732 (100.0)
Age, median (IQR)	41.4 (3.1)	41.5 (3.4)	41.4 (3.3)
Birth year, median (IQR)	1951 (11.5)	1951 (11.5)	1951 (11.5)
Participation year, median (IQR)	1993 (11.2)	1993 (11.4)	1993 (11.2)
Highest attained education^a^	417,109	384,566	801,675
Primary	28.0	24.9	26.5
Secondary	44.3	46.6	45.4
Tertiary	27.7	28.5	28.1
Highest attained income quintile^a^	398,050	378,748	776,798
Quintile 1	7.0	3.6	5.4
Quintile 2	7.9	7.2	7.6
Quintile 3	16.1	16.7	16.4
Quintile 4	26.4	24.4	25.5
Quintile 5	42.4	48.0	45.2
Smoking status (daily)	393,910	364,052	757,962
Never	44.3	36.1	40.4
Former	21.5	27.1	24.2
Current	34.2	36.8	35.5
Smoking intensity (cigarettes/day)^b^	388,957	353,458	742,415
Median (IQR)	5.0 (10.0)	10.0 (15.0)	6.0 (12.5)
Smoking duration (years)^b^	390,408	360,569	750,977
Median (IQR)	5.0 (20.0)	11.0 (22.0)	8.0 (20.0)
Leisure-time physical activity	405,499	372,748	778,247
Category 1	23.9	24.1	24.0
Category 2	57.2	45.2	51.5
Category 3	14.5	23.4	18.7
Category 4	4.5	7.3	5.8
Harmful use of alcohol	110,467	104,581	215,048
No	89.2	76.6	83.1
Yes	10.8	23.4	16.9
Mean alcohol consumption (grams/14 days)	151,803	131,383	283,186
Median (IQR)	28.8 (71.4)	64.5 (100.2)	47.6 (84.1)
Body mass index (kg/m^2^)	409,848	377,462	787,310
Median (IQR)	23.9 (5.0)	25.4 (4.2)	24.7 (4.8)
Underweight	1.8	0.4	1.1
Normal weight	59.6	44.7	52.5
Overweight	27.4	44.4	35.6
Obesity	11.1	10.5	10.8
Blood pressure	368,420	345,653	714,073
Systolic (mmHg), median (IQR)	123.0 (20.0)	133.0 (19.0)	128.0 (21.0)
Diastolic (mmHg), median (IQR)	76.0 (14.0)	80.0 (14.0)	78.0 (14.0)
Blood lipids	329,591	309,130	638,721
Total cholesterol (mmol/L), median (IQR)	5.4 (1.4)	5.8 (1.5)	5.6 (1.4)
Triglycerides (mmol/L), median (IQR)	1.1 (0.8)	1.7 (1.3)	1.4 (1.1)

Data are presented as number of participants or column percentages, unless otherwise indicated. Because of rounding, percentages may not sum up to 100%

*No*. number, *IQR* inter-quartile range.

^a^At study entry.

^b^For current and former smokers.

At study entry, the proportion of missing information ranged from 2.6% (obesity) to 21.0% (hypercholesterolaemia) (Supplementary Fig. S2) but was considerably higher for harmful use of alcohol (73.4%). Further details on missing information in Supplementary Table S5.

### Clustering of NCD risk factors

#### Intersection diagrams

**Figure 3:**
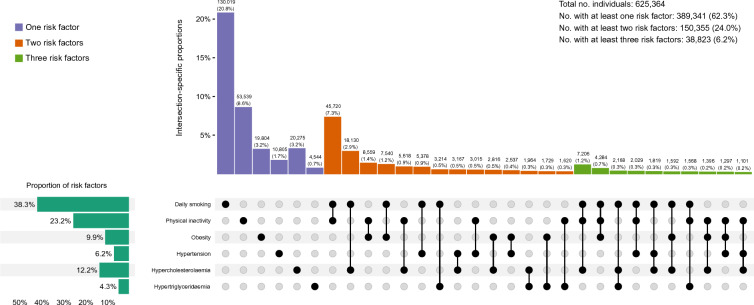
Intersection diagram showing the occurrence and clustering of noncommunicable disease risk factors at study entry (n = 625,364). This shows combinations of noncommunicable disease risk factors in the study sample at study entry. All single risk factor-intersections are shown, as well as the 24 most common combinations of at least two risk factors.

At study entry, 625,364 (77.3%) participants had available information on daily smoking, physical inactivity, obesity, hypertension, hypercholesterolaemia and hypertriglyceridaemia (Fig. 3). Of these, 62.3% had at least one risk factor, 24.0% had at least two risk factors and 6.2% had at least three risk factors. Daily smoking (38.3%), physical inactivity (23.2%), and hypercholesterolaemia (12.2%) were the most prevalent risk factors. The three most common combinations of two risk factors were daily smoking and physical inactivity (7.3%), daily smoking and hypercholesterolaemia (2.9%) and physical inactivity and obesity (1.4%). The most common combination of three risk factors was daily smoking, physical inactivity, and hypercholesterolaemia (1.2%) (Fig. 3). Patterns were similar in women and men, although higher proportions of men had hypercholesterolaemia, hypertension, hypertriglyceridaemia, and multiple risk factors (Supplementary Fig. S3).

The proportion of individuals with at least one risk factor was higher for those included in the periods 1974–1989 (64.8%) and 1990–2004 (63.8%) than for those included later (2005–2019, 56.4%) (Supplementary Fig. S4). From 1974–1989 to 2005–2019, we observed a decrease in daily smoking (from 43.8% to 17.0%) and hypercholesterolaemia (from 16.5% to 9.0%) and an increase in obesity (from 7.1% to 22.8%). Individuals included in 1974–1989 and 1990–2004 were also younger at study entry than those included in 2005–2019 (median [IQR] age 40.6 [2.4], 44.3 [2.4], and 52.2 [21.2] years, respectively). Consequently, the proportion exposed to at least one risk factor was similar or higher among e.g., 40–49-year-olds in 1974–1983 compared to 70–79-year-olds in 2004–2013 (Supplementary Fig. S5).

In the sub-sample that also had data on harmful use of alcohol (n = 78,941), 66.4% had at least one risk factor. The prevalence of harmful use of alcohol was 21.5%, making it the third most prevalent risk factor after daily smoking (26.2%) and physical inactivity (25.8%) (Supplementary Fig. S6).

#### Latent class analysis

**Figure 4:**
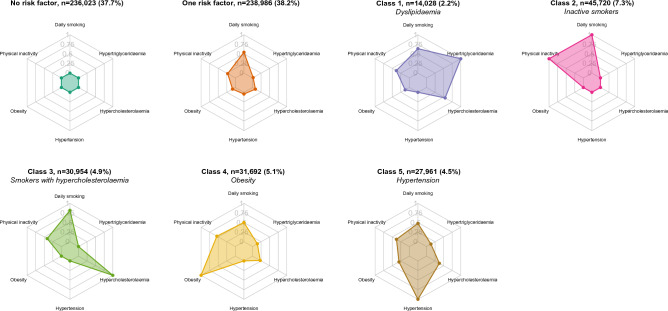
Radar plots showing the distribution of the noncommunicable disease risk factors in each cluster (n = 625,364). For individuals with no or only one risk factor, plots show the proportion of individuals having each risk factor as their single risk factor. For individuals with at least two risk factors, plots show the risk factor distributions of the individuals allocated to each latent class of noncommunicable disease risk factor.

Among individuals with at least two risk factors (n = 150,355), we identified five risk factor clusters. Based on the most prevalent risk factors in each cluster, these were labelled: dyslipidaemia (n = 14,028), inactive smokers (n = 45,720), smokers with hypercholesterolemia (n = 30,954), obesity (n = 31,692) and hypertension (n = 27,961) (Fig. 4). Compared to individuals with no (n = 236,023) and one (n = 238,986) risk factor, individuals in the five risk factor clusters had fewer years of education, lower income, smoked more, and were less physically active (Supplementary Table S7). The cluster labelled hypertension had the lowest proportions of individuals with tertiary education and high income.

#### NCD risk factor trajectories

**Figure 5:**
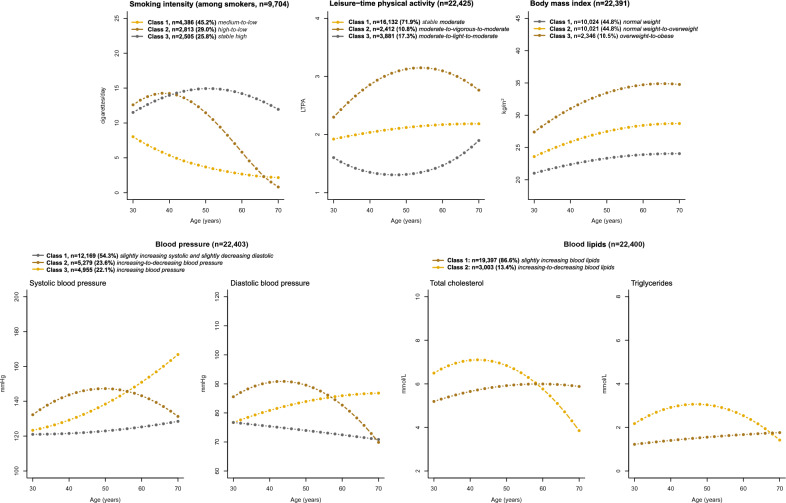
Estimated average trajectories of noncommunicable disease risk factors in each latent class (n = 22,428). For each risk factor and for each latent class, the estimated average trajectory of the risk factor considered among individuals allocated to that class.

Among the 110,512 individuals that participated at least three times, a total of 22,428 (20.3%) individuals participated at least three times between 30 and 70 years of age (90,222 participations in total) (Fig. 5).

Among smokers, three classes of smoking intensity trajectories were identified: medium-to-low (45.2%), high-to-low (29.0%), and stable high (25.8%) (Fig. 5 & Supplementary Fig. S7). At trajectory entry, individuals in the stable high trajectory had the fewest years of education and the highest prevalence of obesity, whereas individuals in the high-to-low trajectory smoked the highest number of cigarettes/day and had the highest prevalence of physical inactivity, but also the lowest prevalence of obesity (Supplementary Table S8).

We identified three classes of LTPA trajectories: stable moderate (71.9%), moderate-to-vigorous-to-moderate (10.8%), and moderate-to-light-to-moderate (17.3%) (Fig. 5 & Supplementary Fig. S8). At trajectory entry, individuals in the moderate-to-vigorous-to-moderate trajectory had the most years of education and highest income, smoked the least and displayed the lowest prevalence of overweight and obesity (Supplementary Table S9). Two thirds of individuals in this trajectory were men.

We identified three classes of BMI trajectories: normal weight (44.8%), normal weight-to-overweight (44.8%), and overweight-to-obese (10.5%). All trajectories were increasing with age (Fig. 5 & Supplementary Fig. S9]). At trajectory entry, individuals in the overweight-to-obese trajectory had fewer years of education, were least physically active and had the highest blood pressure and blood lipid levels (Supplementary Table S10).

We identified three classes of trajectories of combined blood pressure: slightly increasing systolic and slightly decreasing diastolic (54.3%), increasing-to-decreasing blood pressure (23.6%), and increasing blood pressure (22.1%) (Fig. 5 & Supplementary Fig. S10). Individuals in the increasing-to-decreasing blood pressure trajectory had higher blood pressure at trajectory entry compared to the other two, and were more likely to be men, have fewer years of education, and to have overweight or obesity. Individuals in the other two trajectories had similar characteristics, although individuals in the slightly increasing systolic and slightly decreasing diastolic trajectory were more likely to be women and have a BMI < 25 compared to the increasing blood pressure trajectory (Supplementary Table S11).

We identified two classes of trajectories of combined blood lipids: slightly increasing blood lipids (86.6%) and increasing-to-decreasing blood lipids (13.4%) (Fig. 5 & Supplementary Fig. S11). Individuals in the increasing-to-decreasing blood lipids trajectory had higher blood lipids and blood pressure at trajectory entry, were more likely to be men, have fewer years of education, smoke more, and to have overweight or obesity than individuals in the other trajectory (Supplementary Table S12).

## Discussion

We harmonized data on NCD risk factors collected in population-based health studies conducted over a 45-year period to create a single study sample of 808,732 individuals, of which three-quarters had data on all the six key NCD risk factors *daily smoking, physical inactivity, obesity, hypertension*, *hypercholesterolaemia* and *hypertriglyceridaemia* measured at least once. In this sample, about two-thirds were exposed to at least one risk factor, with about one-fourth exposed to at least two risk factors. Daily smoking, physical inactivity, and hypercholesterolaemia were the most prevalent risk factors. We also observed a reduction in the proportion exposed to risk factors after 2004, mainly driven by a marked reduction in daily smoking, and despite a large increase in the prevalence of obesity. Among individuals exposed to at least two risk factors, we identified five distinct clusters of NCD risk factors (dyslipidaemia, inactive smokers, smokers with hypercholesterolaemia, obesity and hypertension), which were characterized by fewer years of education and lower income compared to individuals exposed to less than two risk factors. Lastly, from 30 to 70 years of age, we identified three distinct trajectories of smoking intensity, LTPA, BMI, blood pressure, and two distinct trajectories of blood lipids. Individuals in the trajectories tended to differ in their characteristics, especially across sex, education, and BMI.

Harmonization and pooling of data on individual NCD risk factors to create large datasets are not uncommon,^60,61^ and several of the health studies included in NCDNOR have previously been combined,^62^ but none included all the data sources that we have used. To our knowledge, no study harmonized data on both behavioural and biological risk factors to identify clusters and trajectories of NCD risk factors.

The clustering of NCD risk factors is dependent on which NCD risk factors are considered, how they are measured, the cut-offs used to define risk, and the method used to identify clusters. We chose risk factors based on data-availability and the global strategies for the prevention of NCDs, but also cut-offs believed to represent high NCD risk (higher cut-offs than used e.g. by the WHO)^7^ and reduced risk of misclassification (e.g. due to ‘the white coat syndrome’). Using lower cut-offs causes higher proportions of individuals with clustered risk (e.g. changing cut-offs for hypertension and hypercholesterolemia to grade 1 and 6.2 mmol/L, respectively, the proportion of individuals with clustered risk increases from 24 to 60% [data not shown]).

We found five clusters of risk factors among individuals with at least two of the six risk factors considered. A systematic review on the clustering of behavioural risk factors has, as in the present study, inactive smokers as a common cluster, with prevalence ranging 7–20%.^8^ The only study to date using information on both behavioural and biological risk factors other than obesity, found hypertension, high cholesterol, obesity, and physical inactivity to cluster together.^12^ However, they used different definitions for hypertension, hypercholesterolaemia, and obesity than the present study, yielding much higher overall prevalence of these risk factors. While LCA has been used in other studies, they differ greatly in the numbers and types of risk factors considered, making direct comparisons challenging. To our knowledge this is the largest study describing the clustering of NCD risk factors using information on behavioural and biological risk factors combined, and the first to do so in a Nordic country, making our results unique.

Three studies investigated long-term smoking trajectories extending to age 50 or beyond,^63–65^ two of which modelled smoking intensity and had a follow-up time similar to the present study.^63,64^ Although one study was conducted on women, and both were of much smaller scale, they identified trajectories similar to the ones we identified (medium-to-low, high-to-low, stable high).

In a recent systematic review of studies on physical activity trajectories, 3–5 trajectories were found most common.^66^ Compared to our study, most studies were smaller and had shorter follow-up, but similar to our results, several studies showed that stable trajectories were the most prevalent^66–69^.

We identified three different increasing BMI trajectories, which were similar to those found in other studies conducted in westernized countries of comparatively long follow-up.^70–73^ Other studies less comparable to ours have found as many as nine BMI trajectories^74^.

Several studies have characterized blood pressure trajectories,^75^ but few studies follow individuals over several decades.^76–78^ None modelled systolic and diastolic blood pressure trajectories jointly, and baseline ages were higher^76^ and lower^77^ than in the present study, but all found trajectories comparable to the trajectories we found.

Studies have characterized cholesterol and triglyceride trajectories from childhood to young adulthood ^79^ and middle-age,^80^ and from middle-age to older age.^21^ The latter identified five total cholesterol and four triglyceride trajectories, and similar to our study, the majority of individuals had stable or slightly increasing total cholesterol and triglycerides, and ~ 10–15% had decreasing total cholesterol from age ~ 45.

In the studies that were comparable to ours and reported trajectory-specific participant characteristics, differences were consistently found between trajectories with respect to sex,^63,66–71,76–78,80^ education,^63,66–70,77,78^ and BMI.^61,63,64,66–69,76,78^ This aligns with our study, therefore contributing to the knowledge base on risk factor trajectories and on social inequalities in NCD risk.

The study has several strengths. This is the first large-scale population-based study describing both clustering and trajectories of behavioural and biological NCD risk factors combined. Its prospective design reduces recall bias, and the large sample size provides high statistical power. The study includes adults of all ages covering the entire country, making it uniquely suited to study key NCD risk factors in the Norwegian population. In addition, the data were collected over a 45-year period, with repeated measurements making it possible to investigate risk factor trajectories within individuals over a long time-period. Furthermore, NCD risk factor variables were harmonized between health studies using similar measurement protocols and questionnaires, which limits measurement errors due to harmonization. Lastly, compared to other methods, the LCA and LCMM modelling provide fit-statistics and posterior probabilities, allowing us to investigate model performance.

Nevertheless, we also highlight important limitations. Individuals participating in health studies often differ from the general population, which can result in both over and underestimation of exposure^81,82^.Furthermore, despite high participation rates and the inclusion of studies designed to be at least regionally representative, the health studies were conducted at different time points spanning over five decades. In addition, almost 73% were between 39 and 44 years of age at study entry. This precludes us from generalizing our results to the general adult Norwegian population today. Selection bias may also be exacerbated further by the time window used in LCMMs, restricting the sample to those alive in their 60’s^82^.

Furthermore, due to limited data-availability across studies, we did not include other important risk factors, such as unhealthy diet or hyperglycaemia. Therefore, it is likely that the true prevalence of having at least two NCD risk factors is higher than in our study sample. The clusters and shape of the trajectories may be affected by several factors such as changes in national regulations (e.g. the Norwegian Tobacco Control Act changed several times since 1975)^83^ and medication use (e.g. blood pressure and lipid lowering drugs), data on which were not included in the present study. The study is also subject to measurement errors. Personal education and income were used as proxies for socioeconomic position, which is not ideal, especially for women in earlier cohorts,^84^ blood samples were non-fasting,^47^ and the behavioural risk factors assessed via self-reports, which are subject to well-known biases.^85^ Anthropometrics were also self-reported in HUNT4ST and the 4th Norwegian Counties Study. Lastly, the validity of our results depends on the model specifications chosen. All models were carefully evaluated using several recommended fit-statistics,^57,58^ and performed satisfactorily. While the performance for LCMMs was good, it reflected the irregularity of the measurements. Nonetheless, this can be taken into account in further analyses^59^.

In Norway, the unique national identification number assigned to each resident allows linkage of the individuals included in the health studies to several national mandatory health and administrative registries. Moving forward, this will allow us to investigate how NCD risk factor clusters and trajectories relate to NCD (multi-)morbidity and mortality, and to relate this to life-course socioeconomic circumstances. This will improve the identification of high-risk population groups and help inform future public health policies.

## Conclusion

We described the health studies included in the NCDNOR project and harmonized data on key NCD risk factors. Our results suggest that a large proportion of the adult population in Norway have been exposed to multiple risk factors simultaneously. We identified five distinct clusters of NCD risk factors, where individuals exposed to two or more risk factors were characterized by fewer years of education and lower income compared to individuals exposed to fewer risk factors. Among individuals with repeated measurements, we identified 2–3 long-term trajectories for each risk factor that tended to differ the most across sex, education, and BMI. This study provides important insights into the mechanisms by which NCD risk factors can occur and may help the development of interventions aimed at reducing the NCD burden by targeting multiple risk factors simultaneously.

### Supplementary Information

Supplementary Figures.

Supplementary Information 1.

Supplementary Tables.

## Data Availability

The data that support the findings of this study are available from the Norwegian Health Data Authority and Statistics Norway, but restrictions apply to the availability of these data, which were used under license for the current study, and so are not publicly available. Data are however available from the authors upon reasonable request and with permission of the Norwegian Health Data Authority and Statistics Norway.
