# Supplementary material for: Clustering and trajectories of key noncommunicable disease risk factors in Norway: the NCDNOR project
Source: Sci Rep. 2023 Sep 2;13:14479. doi: 10.1038/s41598-023-41660-x (PMC10475033; doi:10.1038/s41598-023-41660-x)

Supplementary Figures

Knut Eirik Dalene, Simon Lergenmuller et al. Clustering and trajectories of key noncommunicable disease risk factors in Norway – the NCDNOR project

Supplementary Figure S1. Map of Norway showing the geographical distribution of the study sample at study entry (n=808,732). 2

Supplementary Figure S2. Intersection diagram showing the occurrence and clustering of the missing information in the noncommunicable disease risk factors at study entry. 3

Supplementary Figure S3. Intersection diagram showing the occurrence and clustering of noncommunicable disease risk factors at study entry, stratified by sex (n=625,364). 4

Supplementary Figure S4. Intersection diagram showing the occurrence and clustering of noncommunicable disease risk factors at study entry, stratified by inclusion period. 5

Supplementary Figure S5. Heatmap showing the proportion of individuals with at least 1 noncommunicable disease risk factor at study entry by age and inclusion year (n=625,364). 6

Supplementary Figure S6. Intersection diagram showing the occurrence and clustering of noncommunicable disease risk factors at study entry for individuals with information on harmful use of alcohol (n=78,941). 7

Supplementary Figure S7. Estimated average trajectories of smoking intensity (among smokers) in each latent class (n=9,704). 8

Supplementary Figure S8. Estimated average trajectories of leisure-time physical activity (LPTA) in each latent class (n=22,425). 9

Supplementary Figure S9. Estimated average trajectories of body mass index in each latent class (n=22,391). 10

Supplementary Figure S10. Estimated average trajectories of blood pressure in each latent class (n=22,403). 11

Supplementary Figure S11. Estimated average trajectories of blood lipids in each latent class (n=22,400). 12

Supplementary Figure S1. Map of Norway showing the geographical distribution of the study sample at study entry (n=808,732). Map generated using R package "sp" [v1.5-0] based on shapefile data sourced from GADM (2015).

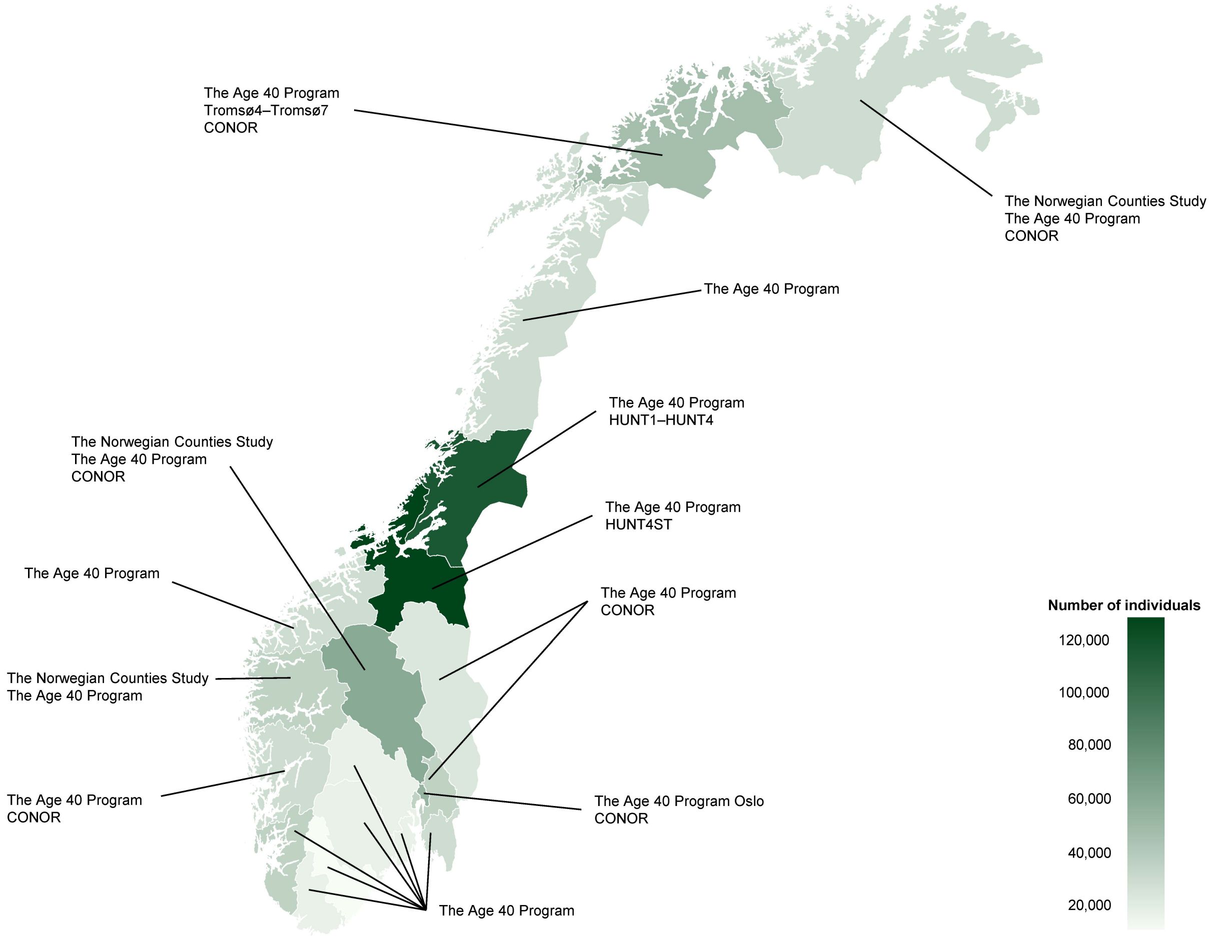

Abbreviations: HUNT, The Trøndelag Health Study; Tromsø, The Tromsø Study; CONOR, Cohort of Norway

Supplementary Figure S2. Intersection diagram showing the occurrence and clustering of the missing information in the noncommunicable disease risk factors at study entry. This shows combinations of missing information in the noncommunicable disease risk factor variables in the study sample at study entry. Only the most common missing information combinations of risk factors are shown. Missing information consists of 77.6% non-measured, non-response (20.7%) and manual cleaning decisions (1.7%).

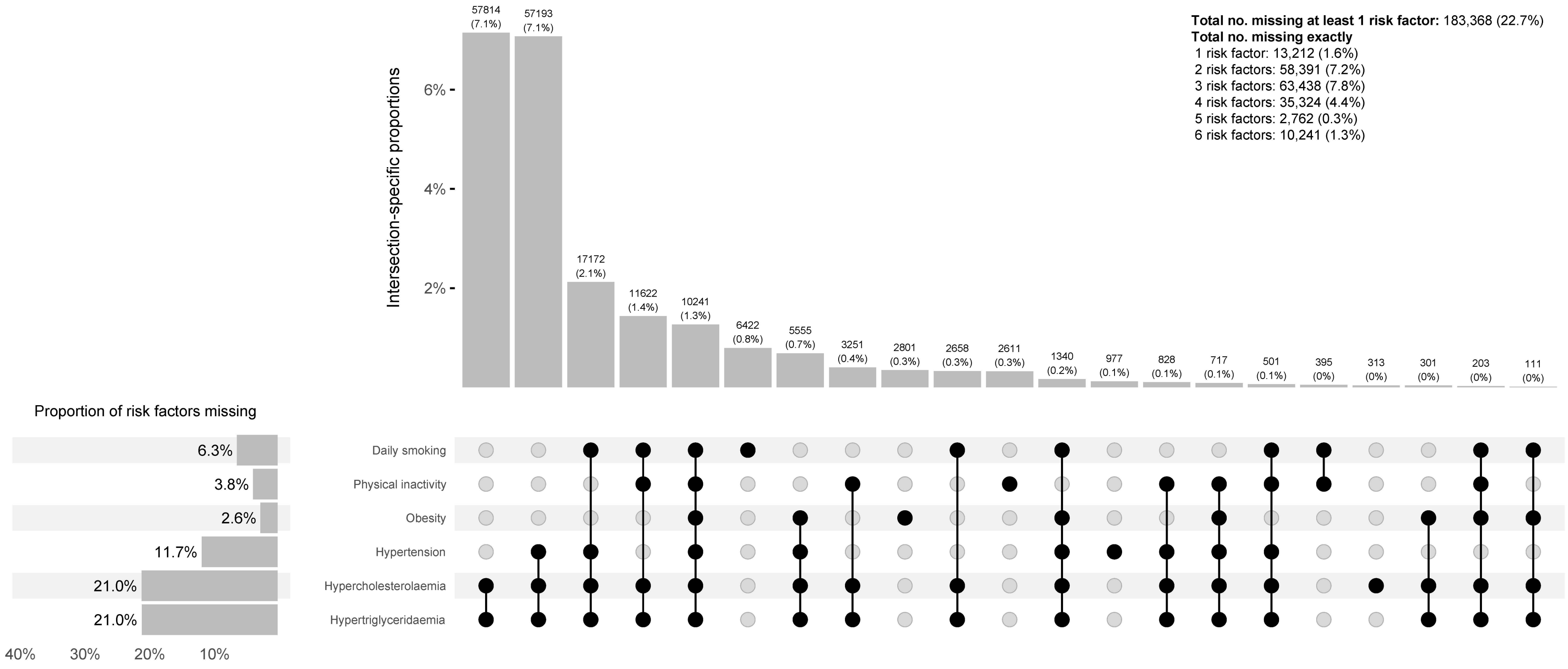

Supplementary Figure S3. Intersection diagram showing the occurrence and clustering of noncommunicable disease risk factors at study entry, stratified by sex (n=625,364). This shows combinations of noncommunicable disease risk factors in the study sample at study entry for women and men. All single risk factor-intersections are shown, as well as the 24 most common combinations of at least two risk factors.

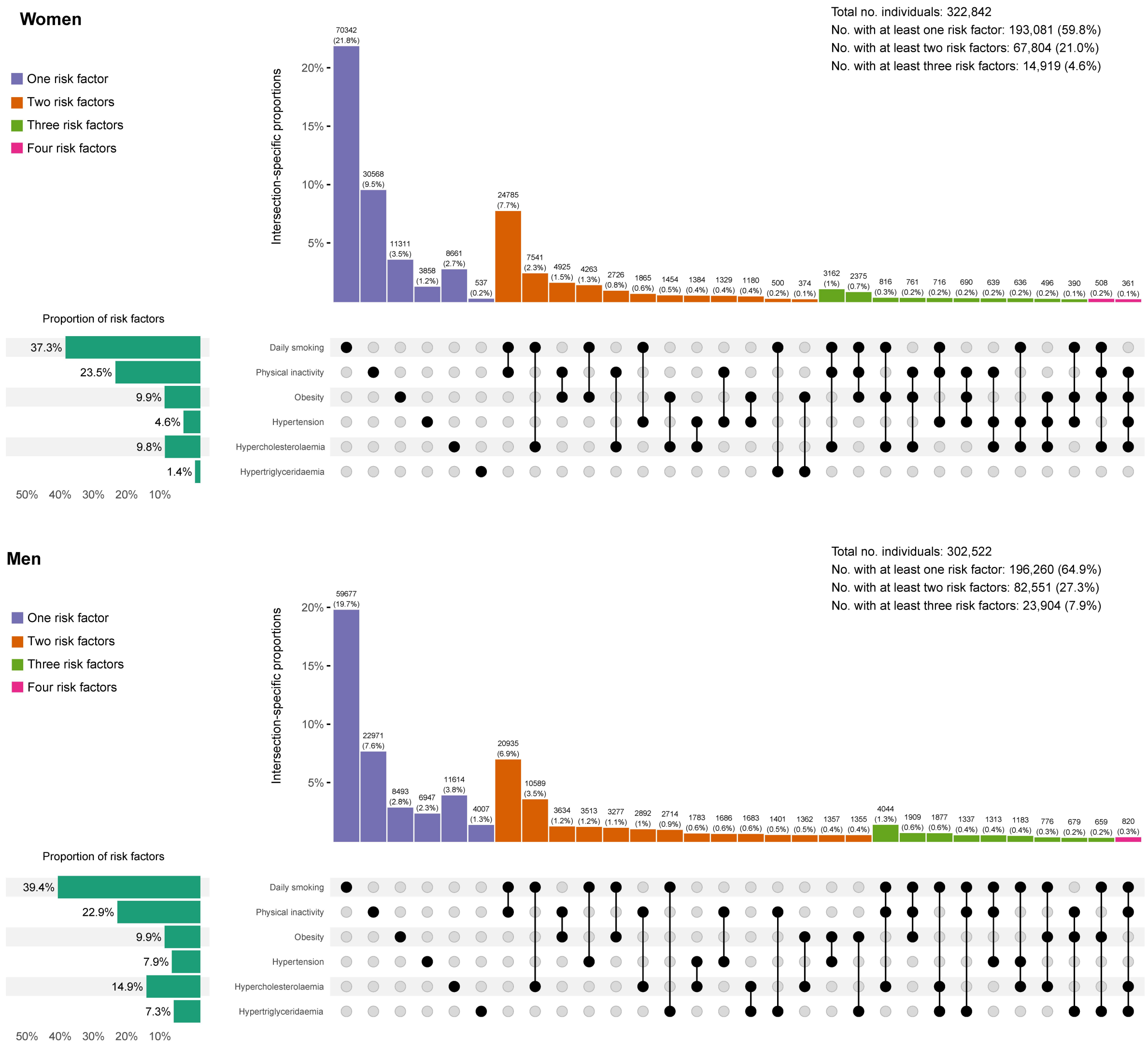

Supplementary Figure S4. Intersection diagram showing the occurrence and clustering of noncommunicable disease risk factors at study entry, stratified by inclusion period. This shows combinations of noncommunicable disease risk factors in the study sample for individuals with a participation in the period 1974-1989, 1990-2004 or 2005-2019 (only the first participation in the period counts). All single risk factor-intersections are shown, as well as the 24 most common combinations of at least two risk factors. Violin plots (lines represent median and quartiles), show the birth year and age distributions of the individuals included in each time-period Individuals may contribute to more than one period, but never twice to the same period.

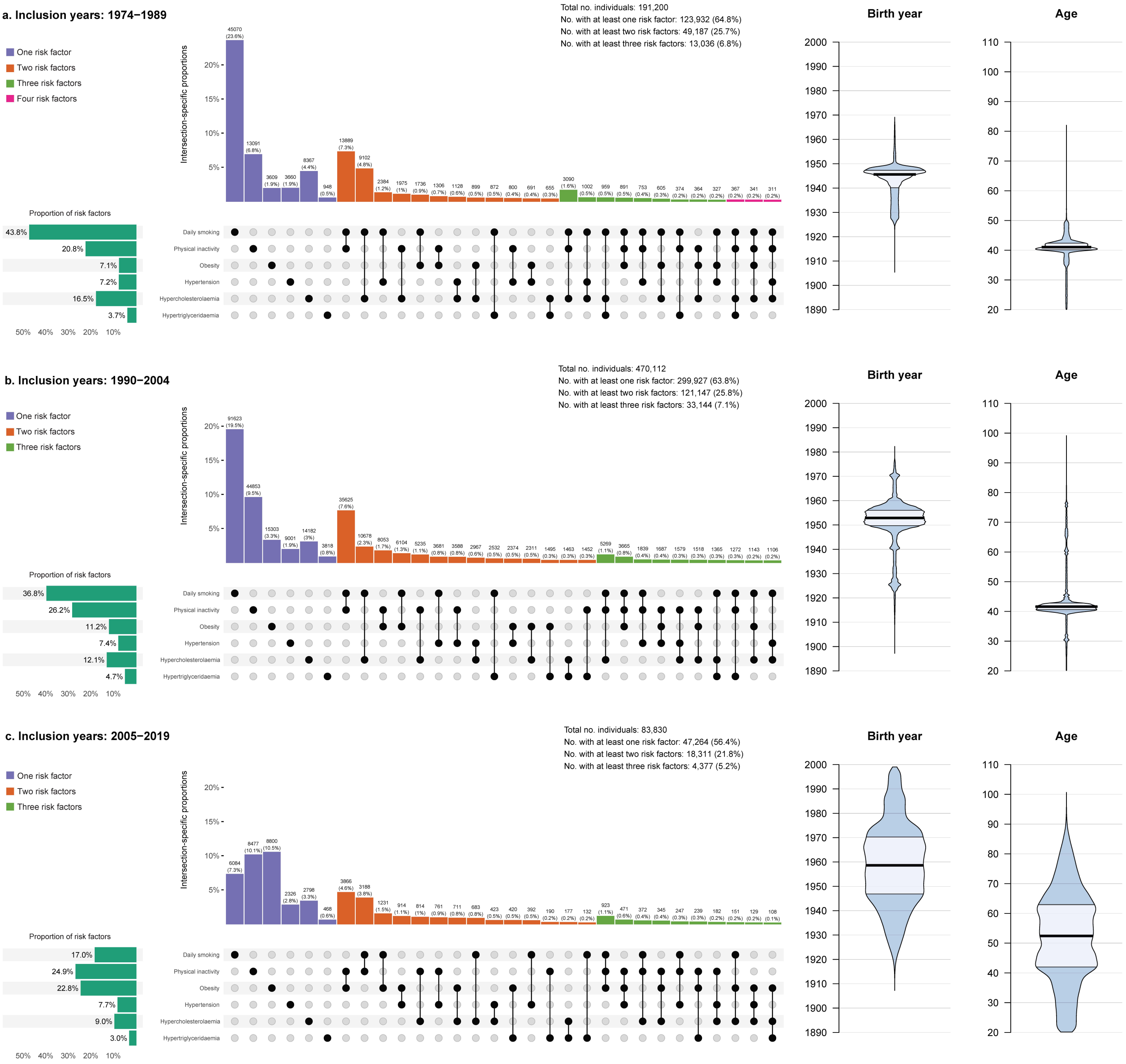

Supplementary Figure S5. Heatmap showing the proportion of individuals with at least 1 noncommunicable disease risk factor at study entry by age and inclusion year (n=625,364). Each square corresponds to a specific age-inclusion window (10-year age period and 10-year inclusion period). The figure shows the proportion of individuals with at least one risk factor at study entry in each age-inclusion window.

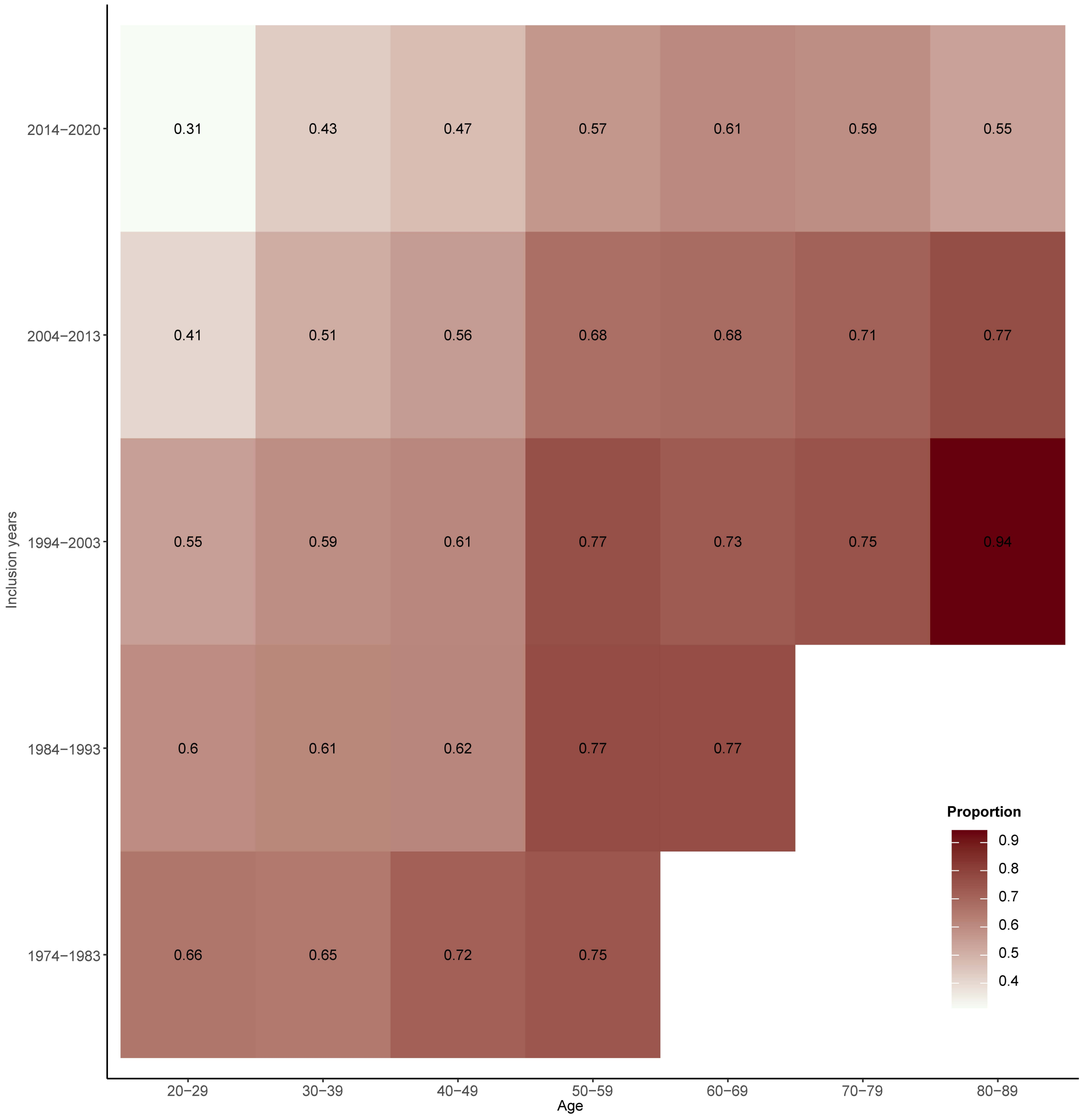

Supplementary Figure S6. Intersection diagram showing the occurrence and clustering of noncommunicable disease risk factors at study entry for individuals with information on harmful use of alcohol (n=78,941). This shows combinations of noncommunicable disease risk factors in the study sample for individuals that also had information on harmful use of alcohol (in addition to the other risk factors). All single risk factor-intersections are shown, as well as the 23 most common combinations of at least two risk factors.

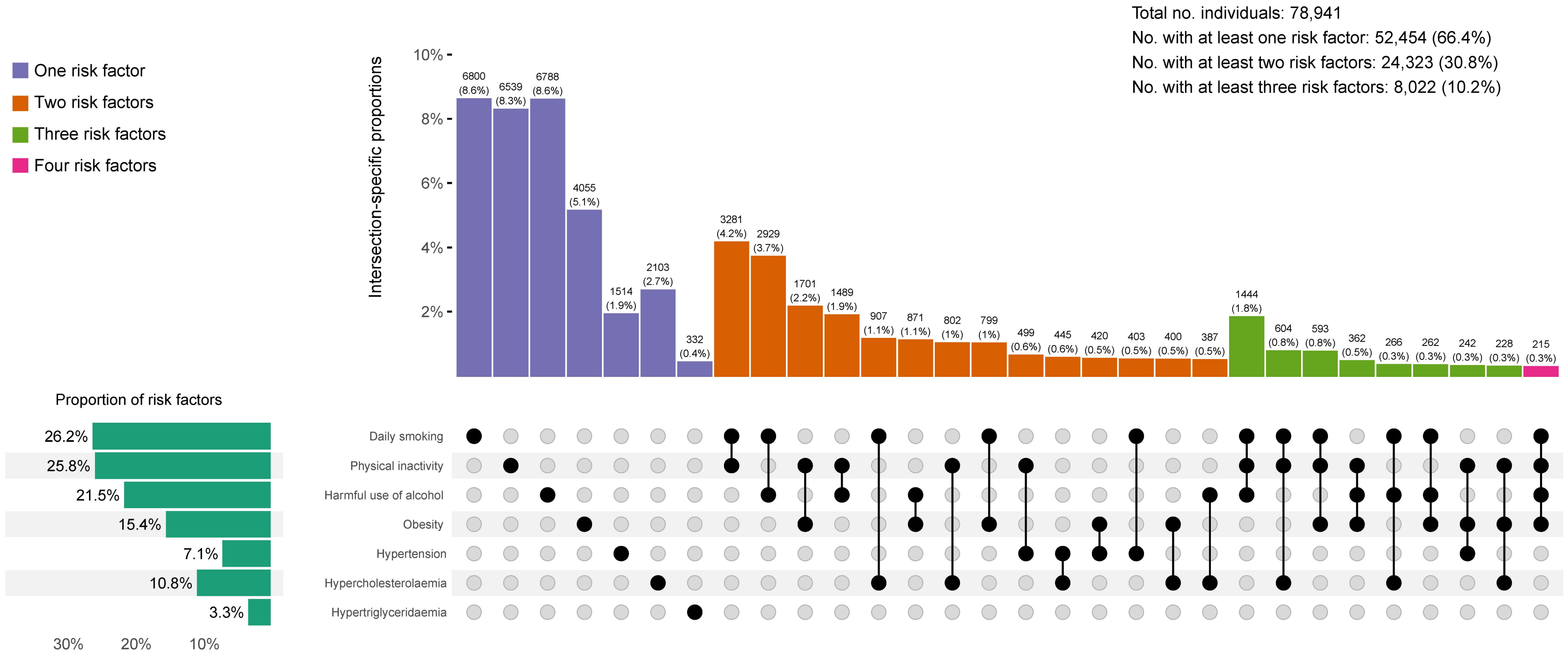

Supplementary Figure S7. Estimated average trajectories of smoking intensity (among smokers) in each latent class (n=9,704). Panel a: For each latent class, the estimated average trajectory of smoking intensity among individuals allocated to that class. Panels b, c, and d: The estimated trajectory for each class separately (dotted lines) with a random sample of 100 observed individual trajectories (solid lines) displayed in the background, picked among individuals allocated to each class.

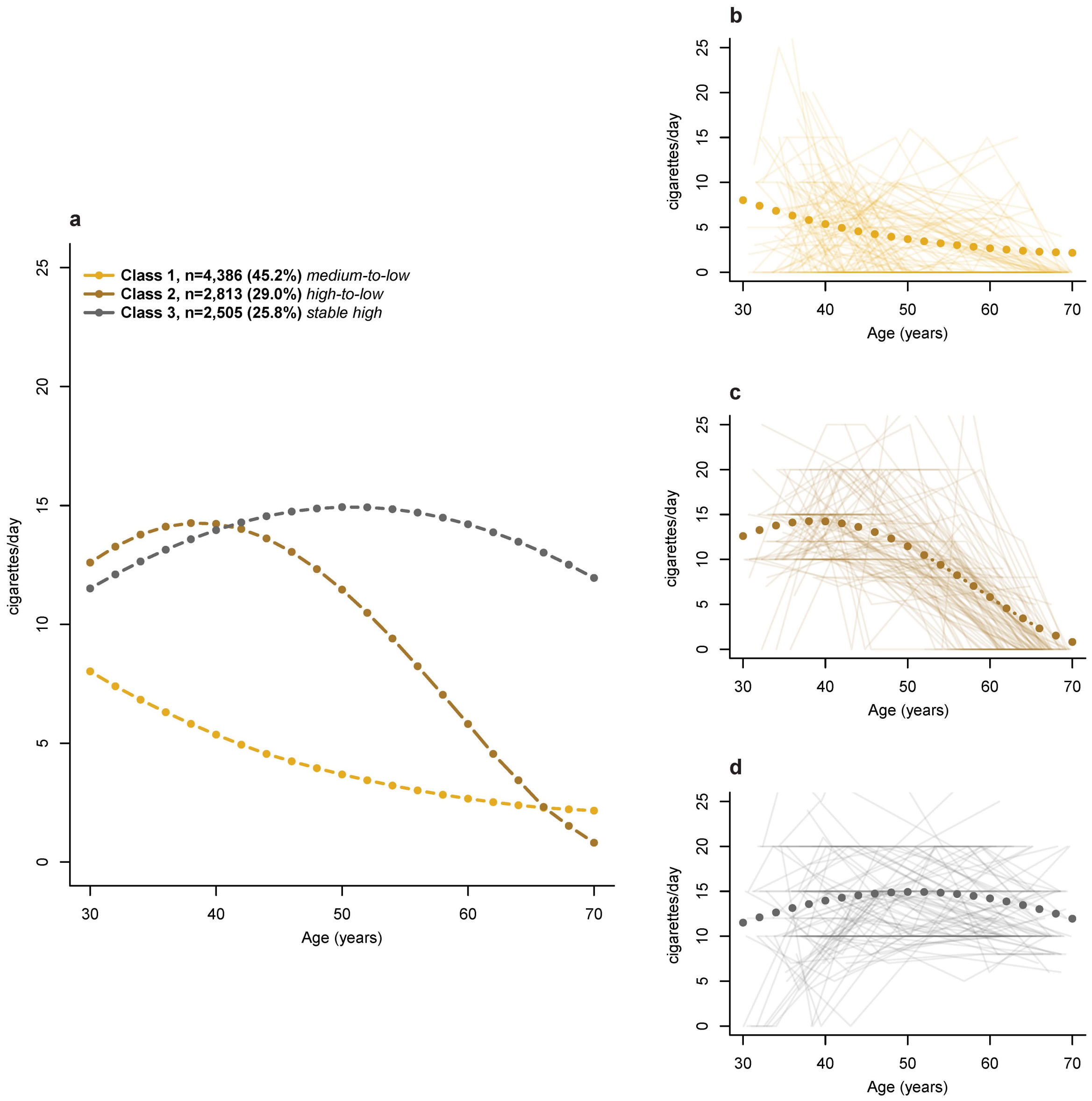

Supplementary Figure S8. Estimated average trajectories of leisure-time physical activity (LPTA) in each latent class (n=22,425). Panel a: For each latent class, the estimated average trajectory of leisure-time physical activity among individuals allocated to that class. Panels b–d: The estimated trajectory for each class separately (dotted lines) with a random sample of 100 observed individual trajectories (solid lines) displayed in the background, picked among individuals allocated to each class.

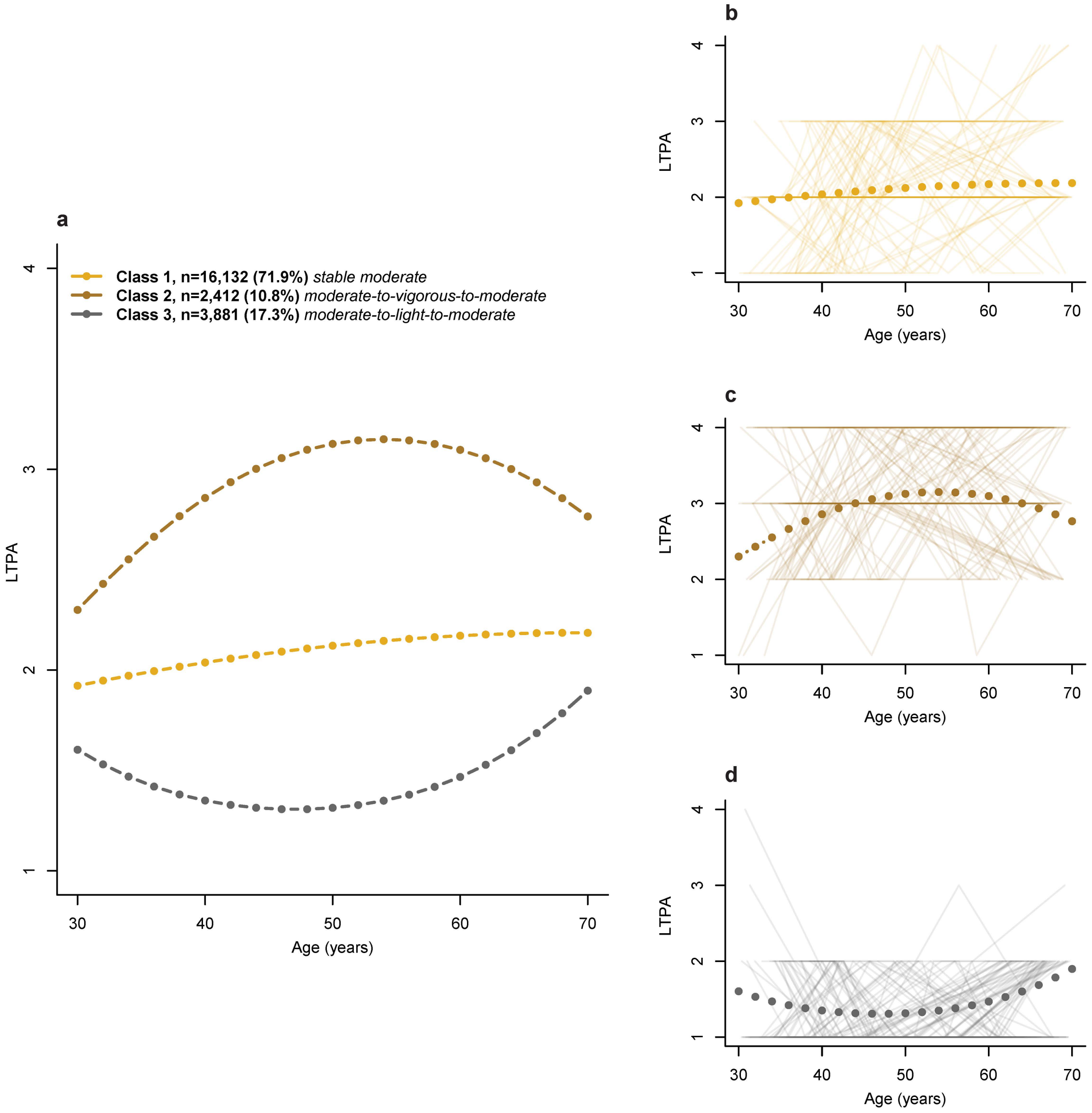

Supplementary Figure S9. Estimated average trajectories of body mass index in each latent class (n=22,391). Panel a: For each latent class, the estimated average trajectory of body mass index among individuals allocated to that class. Panels b–d: The estimated trajectory for each class separately (dotted lines) with a random sample of 100 observed individual trajectories (solid lines) displayed in the background, picked among individuals allocated to each class.

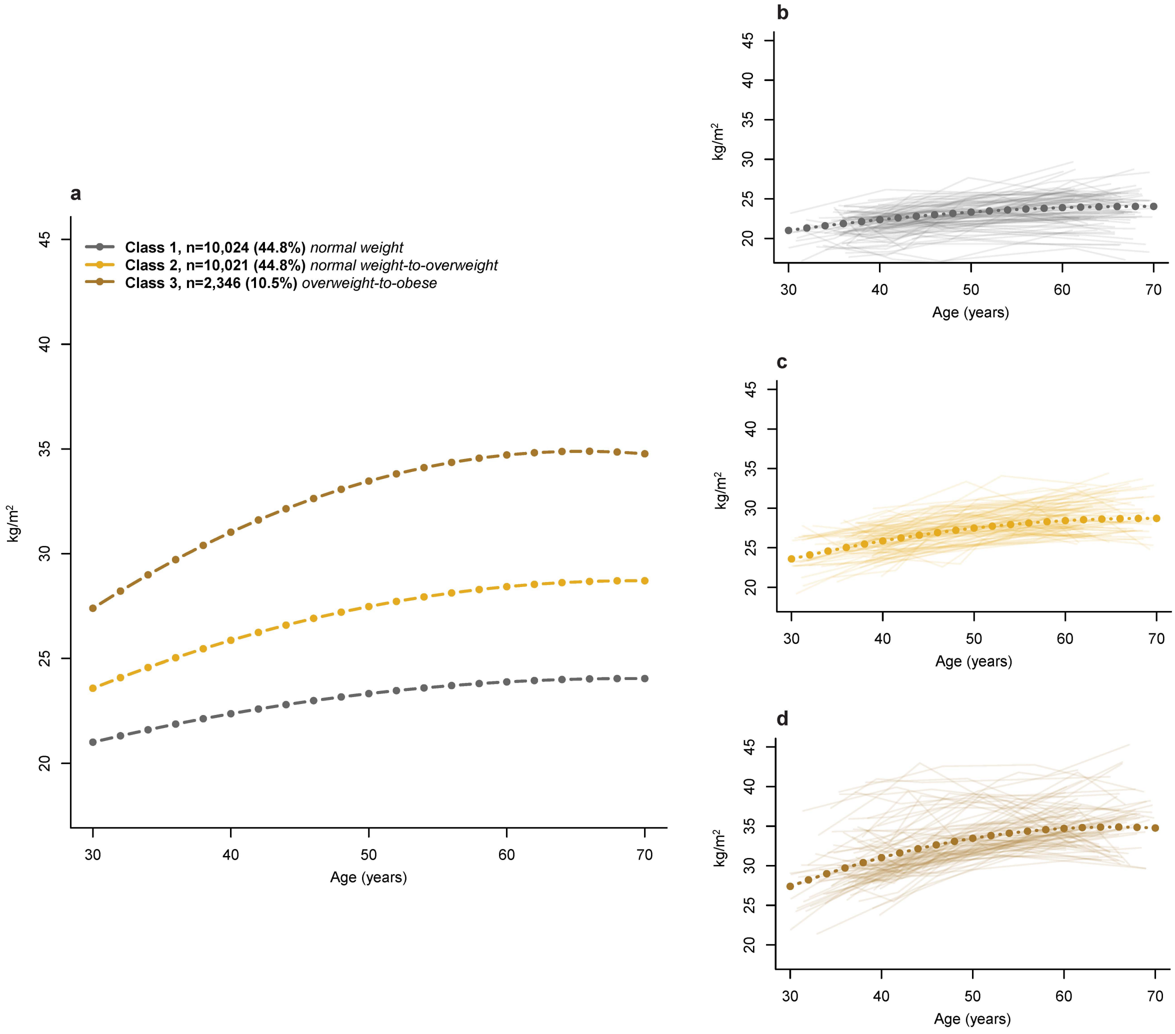

Supplementary Figure S10. Estimated average trajectories of blood pressure in each latent class (n=22,403). Panels a and e: For each latent class, the estimated average trajectory of systolic blood pressure and diastolic blood pressure among individuals allocated to that class. Panels b–d and f–h: The estimated trajectories for each class separately (dotted lines) with a random sample of 100 observed individual trajectories (solid lines) displayed in the background, picked among individuals allocated to each class.

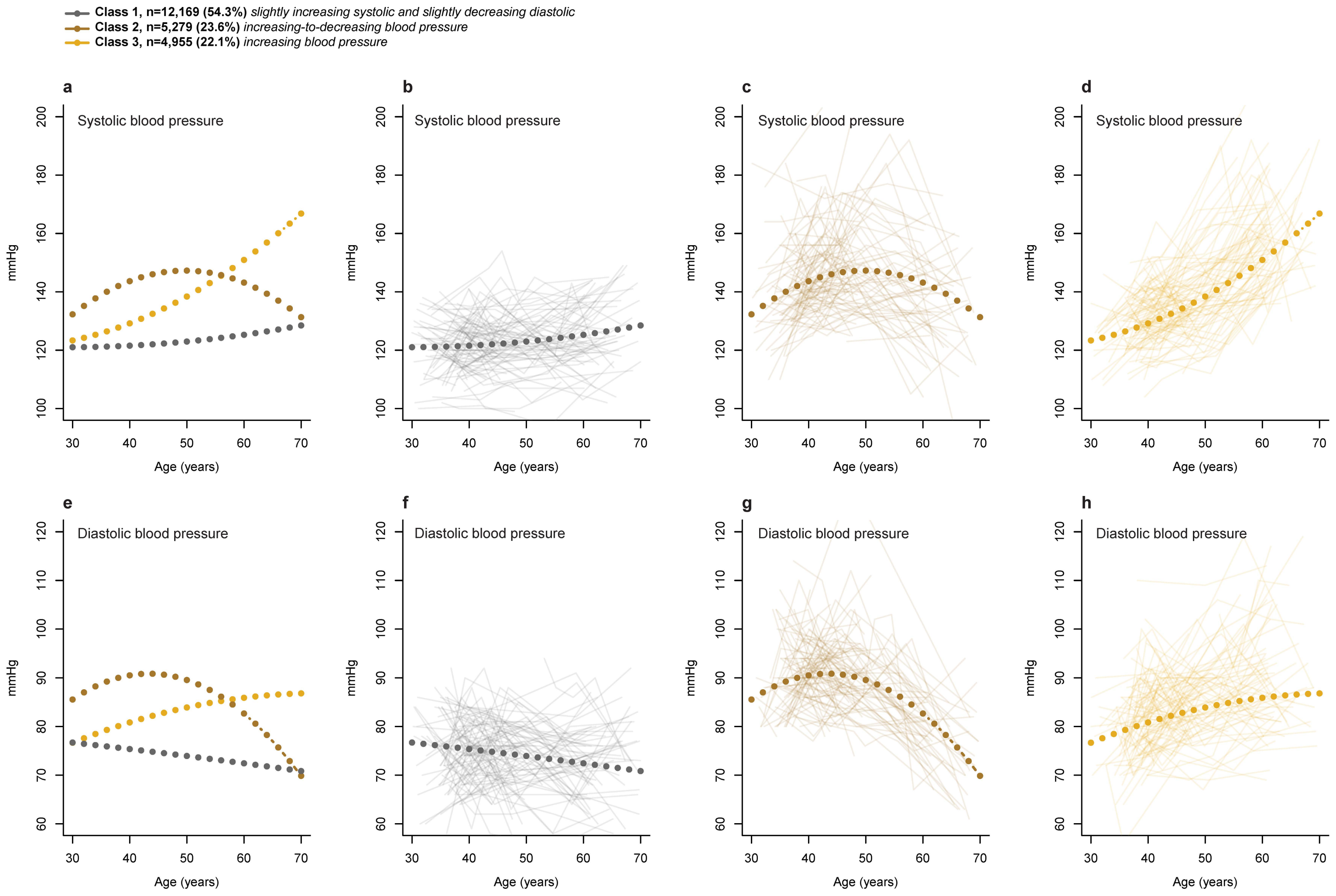

Supplementary Figure S11. Estimated average trajectories of blood lipids in each latent class (n=22,400). Panels a and d: For each latent class, the estimated average trajectory of total cholesterol and triglycerides among individuals allocated to that class. Panels b–c and e–f: The estimated trajectory for each class separately (dotted lines) with a random sample of 100 observed individual trajectories (solid lines) displayed in the background, picked among individuals allocated to each class.

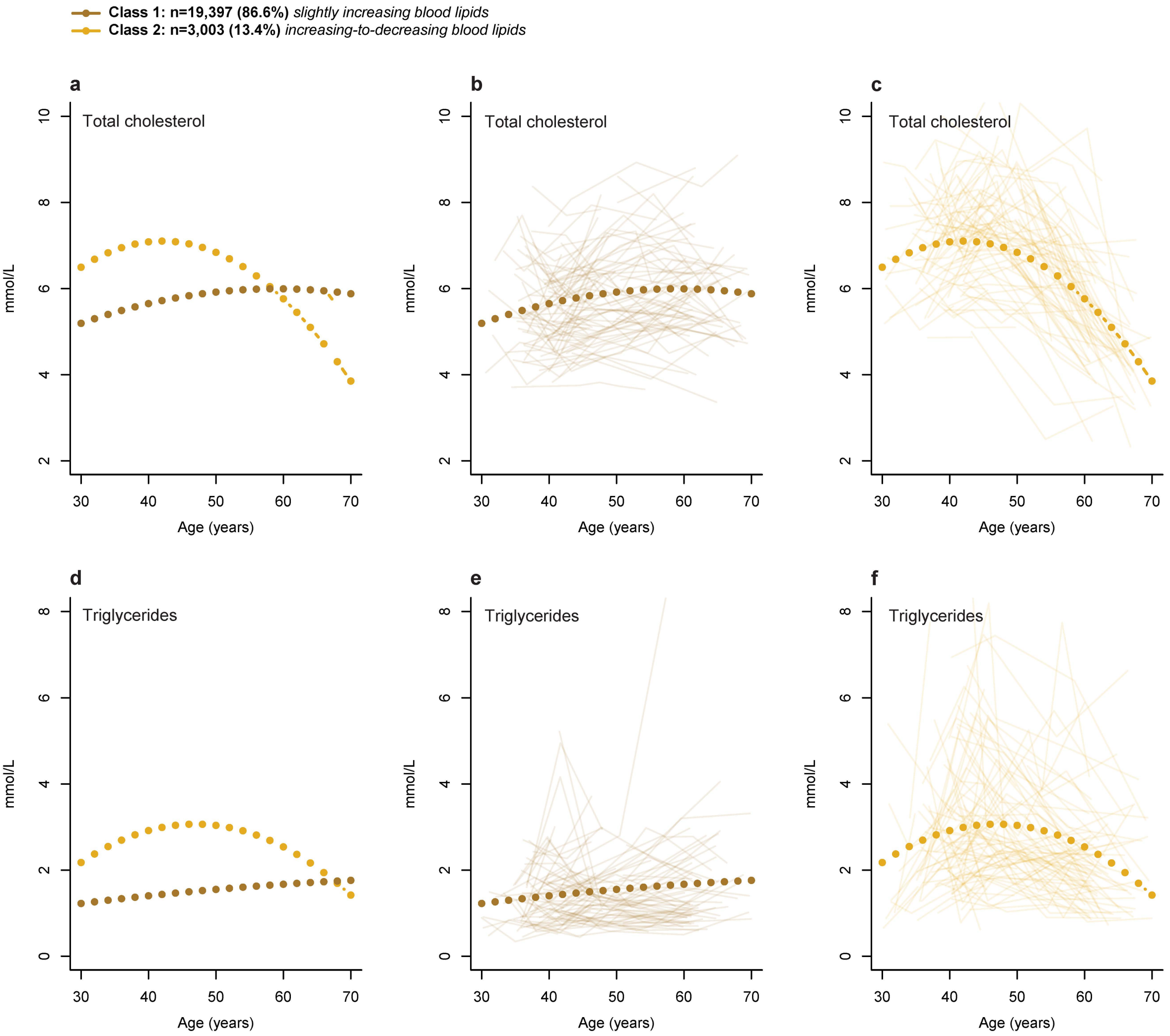

Supplement: Supplementary file 1 — Supplementary Figures. [file 41598_2023_41660_MOESM1_ESM.pdf]
