# Supplementary material for: Clustering and trajectories of key noncommunicable disease risk factors in Norway: the NCDNOR project
Source: Sci Rep. 2023 Sep 2;13:14479. doi: 10.1038/s41598-023-41660-x (PMC10475033; doi:10.1038/s41598-023-41660-x)
Supplement: Supplementary file 2 — Supplementary Information 1. [file 41598_2023_41660_MOESM2_ESM.pdf]

Supplementary Methods

Knut Eirik Dalene, Simon Lergenmuller et al. Clustering and trajectories of key noncommunicable disease risk factors in Norway – the NCDNOR project

Supplementary Methods 1: Identification of NCD risk factor clusters using Latent Class Analysis....... 2

Supplementary Methods 2: Identification of trajectories of noncommunicable disease risk factors using Latent Class Mixed Models 5

Supplementary Methods 3: Overview of sensitivity analyses 10

Supplementary Methods 1: Identification of NCD risk factor clusters using Latent Class Analysis

We assume that there exist ***K*** underlying, unobserved groups of individuals (latent classes) that cluster with respect to their patterns of risk factors. The objective is to identify the optimal number (***K***) of classes. We use latent class analysis, a method adapted for the analysis of multivariate categorical data.[1,2]

LCA models are finite mixture models, and two parameters are estimated: the class-conditional probability that an observation in class *k* produces a specific outcome on a specific variable, and the "prior" probability of class membership. The LCA model allows for the inclusion of covariates, but for simplicity, and to avoid interpretability issues, we use the "unconditional" LCA model, without covariates.

We were interested in the clustering of NCD risk factors, implying the occurrence of minimum two risk factors simultaneously. Therefore, we restricted the LCA to the sample of individuals with ≥2 risk factors. To find the optimal number of classes, we fitted models with increasing number of classes, starting with the 1-class model. We evaluated each model in terms of: quality of the model fit (Akaike Information Criterion [AIC], Bayesian Information Criterion [BIC]), classification power (model entropy, and posterior probability tables), and relevance of the classes.[1,2] To ensure convergence to a global maximum, we ran each model 100 times with different starting values.[3] We tested 1-6 models, and the model with 5 classes was found to be optimal (Table A below). Once the optimal number of classes was identified, we allocated individuals to the class for which they had the largest posterior probability (modal assignment).

Table A. Akaike Information Criterion (AIC), Bayesian Information Criterion (BIC) and entropy for the models with 1-6 classes.

	AIC	BIC	Entropy	No. in smallest class (%)*
**1-class model**	1024451	1024510	1.000	150,355 (100)
**2-class model**	972514	972643	0.775	45,720 (30.4)
**3-class model**	946538	946737	0.794	39,030 (26.0)
**4-class model**	931630	931898	0.916	27,961 (18.6)
**5-class model**	915607	915945	0.979	14,028 (9.3)
**6-class model**	903834	904241	0.977	5,618 (3.7)

*classes allocated by modal assignment. Percentages are relative to the sample of individuals with ≥2 risk factors (n=150,355)

AIC/BIC

A model with lower AIC/BIC will typically represent a model with better fit. Loosely speaking, the difference between AIC and BIC is that BIC will penalize models with higher number of parameters, and BIC has been shown to outperform AIC for class selection.[4] Following these criteria, the 6-class model seems to perform best, although both AIC and BIC were linearly decreasing. In that case, we may rely on the other criteria described below.

Entropy

Entropy here is a number between 0 and 1 that assesses the discriminatory power of the latent class structure. The closer to one, the better the discrimination. The 5-class model had the best entropy. Note however that high entropy may result from overfitting, which can lead to classes containing very few individuals.[5,6]

Class distribution

As the number of classes increases, the percentage of individuals in each class decreases. We may discard models for which the percentage of individuals in the smallest class is very low. A sometimes

used cutoff, though somewhat arbitrary, is a smallest class proportion of 5%.[7] From Table A, we see that the smallest class in the 6-class model contained 3.6% of individuals, while the lowest percentage for the 5-class model was higher (9.3%). Based on this criterion, the 5-class model was best.

Posterior probability classification tables for the 5-class model

For each individual, the estimated posterior class membership probabilities can be obtained. By allocating individuals to the class for which they had the largest posterior probability (modal assignment), we can compute the matrix of mean posterior probabilities to belong to each class among individuals that were *a posteriori* allocated to that class (Table B below). Diagonal elements closer to one and off-diagonal elements closer to zero indicate better classification. Perfect classification translates into diagonal elements equal to one and off-diagonal elements equal to zero.

Table B. Posterior classification table for the five classes of NCD risk factor cluster*

	**Mean of the posterior probabilities of belonging to each class of NCD risk factor cluster**				
	**Class 1**	**Class 2****	**Class 3****	**Class 4**	**Class 5**
**Class 1**	0.946	0.007	0.000	0.046	0.000
**Class 2****	0.000	1.000	0.000	0.000	0.000
**Class 3****	0.000	0.000	1.000	0.000	0.000
**Class 4**	0.049	0.000	0.000	0.951	0.000
**Class 5**	0.000	0.007	0.000	0.000	0.993

* Class 1, dyslipidaemia; Class 2, inactive smokers; Class 3, smokers with hypercholesterolemia; Class 4, obesity; Class 5, hypertension
** Class 2 and 3 had perfect classifications. Class 2 consisted of all inactive smokers. Class 3 consisted of all smokers with hypercholesterolaemia, inactive smokers with hypercholesterolaemia and inactive individuals with hypercholesterolaemia.

Table B indicates near perfect classification for the 5-class model.

Relevance of the classes

The relevance of the classes was visually assessed by plotting spider diagrams for each model. By comparing these to the intersection diagram we could assess to which extent the LCA models capture the clustering of NCD risk factors. This is rather subjective, and the optimal model was therefore selected primarily based on the other criteria.

References

1. Linzer DA, Lewis JB. poLCA: An R package for polytomous variable latent class analysis. *Journal of statistical software* 2011; **42**: 1-29.
2. Nguefack HLN, Pagé MG, Katz J, et al. Trajectory modelling techniques useful to epidemiological research: A comparative narrative review of approaches. *Clin Epidemiol* 2020; **12**: 1205.
3. van de Schoot R, Sijbrandij M, Winter SD, Depaoli S, Vermunt JK. The GRoLTS-Checklist: Guidelines for Reporting on Latent Trajectory Studies. *Structural Equation Modeling: A Multidisciplinary Journal* 2017; **24**(3): 451-67.
4. Morgan GB, Hodge KJ, Baggett AR. Latent profile analysis with nonnormal mixtures: A Monte Carlo examination of model selection using fit indices. *Computational Statistics & Data Analysis* 2016; **93**: 146-61.
5. Sinha P, Calfee CS, Delucchi KL. Practitioner's Guide to Latent Class Analysis: Methodological Considerations and Common Pitfalls. *Critical Care Medicine* 2021; **49**(1): e63-e79.

6. Sinha P, Calfee CS, Delucchi KL. Practitioner's guide to latent class analysis: methodological considerations and common pitfalls. *Crit Care Med* 2021; **49**(1): e63.
7. Shanahan L, Copeland WE, Worthman CM, Erkanli A, Angold A, Costello EJ. Sex-differentiated changes in C-reactive protein from ages 9 to 21: The contributions of BMI and physical/sexual maturation. *Psychoneuroendocrinology* 2013; **38**(10): 2209-17.

Supplementary Methods 2: Identification of trajectories of noncommunicable disease risk factors using Latent Class Mixed Models

For each risk factor *j, w*e assume that there exist K_j underlying, unobserved groups of individuals (latent classes) that follow class-specific trajectories of the risk factor. The objective is to identify for each risk factor *j*, the optimal number (K_j) of classes. We use latent class mixed models (LCMMs),[1-3] also referred to as growth mixture model.[1]

Using LCMM we can identify trajectories for one risk factor at the time. Sometimes, it is meaningful to consider several risk factors simultaneously. In this paper, this was the case for systolic and diastolic blood pressure, as well as for total cholesterol and triglycerides. This was done using multivariate LCMM.[4] Because the estimation procedures are similar to "univariate" LCMM, we do not distinguish the two in the following, and refer to both as LCMMs.

To best describe the dynamic aspect of the cohort, we chose to estimate trajectories of risk factors for those with at least 3 participations in the time window from 30 years of age to 70 years of age. Restricting the sample to a minimum of 3 participations facilitates the capture of curvatures in the longitudinal measurements. To further improve the quality of the trajectories, we restricted the analyses to individuals with at least one measurement in the period 30-40 years, and at least one measurement in the period 60-70 years. This restriction is important to avoid measurements from specific age-spans to dominate the trajectories (eg too many measurements at the start, and/or too few at the end of the time window).

LCMMs consist of a first model for the probability of belonging to each of the classes, and a second model for the mean lifetime risk factor-trajectory in each class. These two models form a finite mixture model. The model for the probability of belonging to each of the classes was the same for all risk factors: a multinomial logistic regression with class-specific intercept. The model for the mean lifetime risk factor-trajectories was also the same for all risk factors, and only included "age", which was modelled as a second order polynomial for flexibility. LCMMs allow for the inclusion of covariates, but for simplicity, and to avoid interpretability issues, we use the "unconditional" LCMM models.

For each risk factor, we evaluated whether the inclusion of a random intercept (allowing observations to be correlated in time) improved the fit. For smoking intensity, the distribution of cigarettes/day was skewed (Figure A below), and a normalizing transformation was used. We used I-spline transformation (more flexible than log-transformation). To do so, we need to specify a series of knots. The simplest combinations of 3-equidistant knots was chosen. LTPA is a categorical variable, and LCMMs allow the handling of such variables using a cumulative probit model. Except for triglycerides, the other variables (body mass index [BMI], systolic blood pressure, diastolic blood pressure, total cholesterol) very close to normally distributed (Figure A below). Although the distribution of triglycerides was skewed, a normalizing transformation did not change the results and was not necessary.

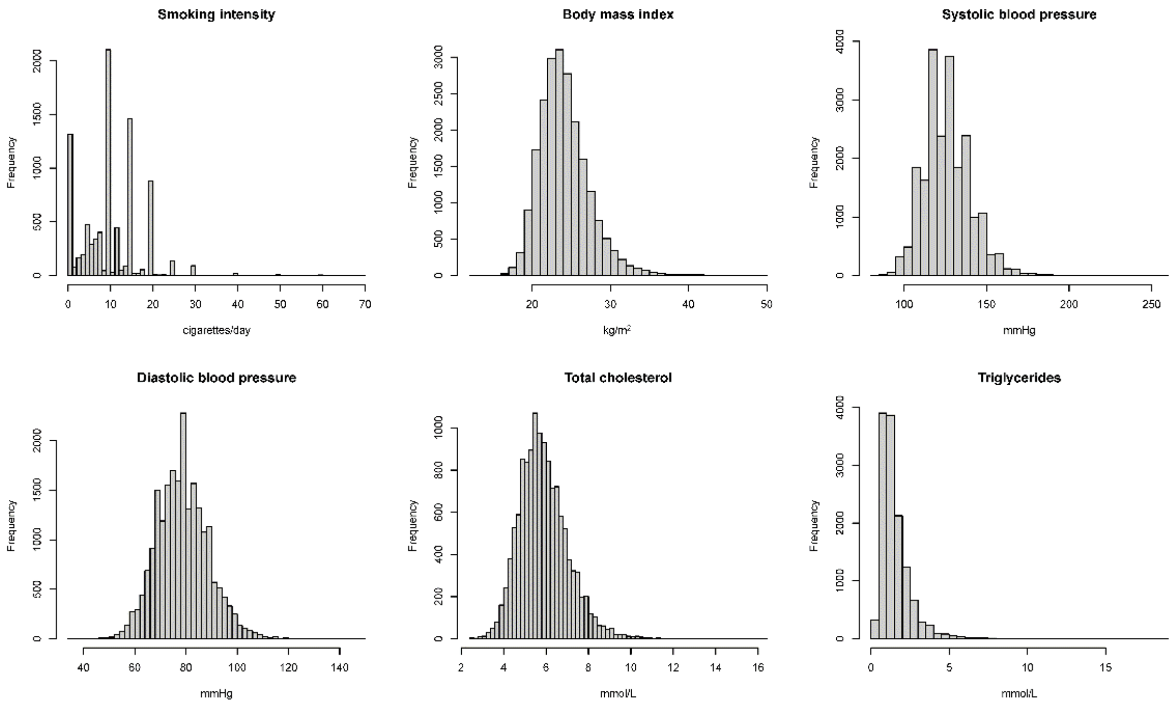

Figure A. Histograms showing the distribution of the risk factors at trajectory entry.

To find the optimal number of classes, for each risk factor, we fitted models with increasing number of classes, starting with the 1-class model. We evaluated each model in terms of: quality of the model fit (Akaike Information Criterion [AIC], Bayesian Information Criterion [BIC]), classification power (model entropy, and posterior probability tables), and relevance of the classes.[5-8] To ensure convergence to the global maximum, we used a grid of 100 initial values.[9] We tested 1-5 models, and for simplicity, we only present a summary table of the best model for each risk factor (Table A below). Once the optimal number of classes was identified, we allocated individuals to the class for which they had the largest posterior probability (modal assignment).

Table A. Summary table of the "best" models for each risk factors

Risk factor	No. of classes	Transformation	Random intercept?	Entropy	No. in smallest class (%)*
Smoking intensity	3	3 equidistant splines	Yes	0.651	2,505 (25.8%)
LTPA	3	Cumulative probit model	No	0.630	2,412 (10.8%)
BMI	3	-	No	0.846	2,346 (10.5%)
Systolic blood pressure**	3	-	Yes	0.640	4,955 (22.1%)
Diastolic blood pressure**					
Total cholesterol**	2	-	Yes	0.789	3,003 (13.4%)
Triglycerides**		-			

*classes allocated by modal assignment.
**estimated by multivariate LCMM

Selected details on the model selection

Entropy

Entropy here is a number between 0 and 1 that assesses the discriminatory power of the latent class structure. The closer to one, the better the discrimination. The entropy was good for BMI and blood lipids and performed worse for smoking intensity, LTPA and blood pressure. Lower entropy might indicate bad fit but may also reflect the irregularity of the measurements: participations to the health studies did not happen at regular intervals, and not all individuals had the same number of participations. This creates "holes" in the longitudinal data leading to more uncertainty in the

classification (lower posterior probabilities), and the resulting entropy (which is a function of the posterior probabilities) will typically be lower. This can be taken into account when relating the classes to an outcome in the future.[8] In general, the "best" models usually were a result of a balance between model entropy and class distribution.

Class distribution

Increasing the number of classes in general led to poorer class distributions (ie very few individuals in the smallest class, typically <5%). When choosing between two models with similar entropy, a model with better class distribution was usually considered best.

Posterior probability classification tables for each model

For each individual, the estimated posterior class membership probabilities can be obtained. By allocating individuals to the class for which they had the largest posterior probability (modal assignment), we can compute the matrix of mean posterior probabilities to belong to each class among individuals that were *a posteriori* allocated to that class (Tables B1-B5 below). Diagonal elements closer to one and off-diagonal elements closer to zero indicate better classification. Perfect classification translates into diagonal elements equal to one and off-diagonal elements equal to zero.

Table B1. Posterior classification table for the three classes of smoking intensity trajectories

	**Mean of the posterior probabilities of belonging to each class of smoking intensity trajectory**		
	**Class 1,** Medium-to-low	**Class 2,** High-to-low	**Class 3,** Stable high
**Class 1,** Medium-to-low	0.880	0.097	0.023
**Class 2,** High-to-low	0.133	0.799	0.068
**Class 3,** Stable high	0.049	0.137	0.814

Table B2. Posterior classification table for the three classes of leisure-time physical activity trajectories

	**Mean of the posterior probabilities of belonging to each class of LTPA trajectory**		
	**Class 1,** Stable moderate	**Class 2,** Moderate-to-vigorous-to moderate	**Class 3,** Moderate-to-light-to-moderate
**Class 1,** Stable moderate	0.847	0.066	0.087
**Class 2,** Moderate-to-vigorous-to moderate	0.211	0.789	0.000
**Class 3,** Moderate-to-light-to-moderate	0.211	0.000	0.789

Abbreviations: LTPA, leisure-time physical activity

Table B3. Posterior classification table for the three classes of body mass index trajectories

	**Mean of the posterior probabilities of belonging to each class of BMI trajectory**		
	**Class 1,** Normal weight	**Class 2,** Normal weight-to-overweight	**Class 3,** Overweight-to-obese
**Class 1,** Normal weight	0.933	0.067	0.000
**Class 2,**	0.066	0.919	0.015

Normal weight-to-overweight			
**Class 3,** Overweight-to-obese	0.000	0.053	0.947

Abbreviations: BMI, body mass index

Table B4. Posterior classification table for the three classes of blood pressure trajectories

	**Mean of the posterior probabilities of belonging to each class of blood pressure trajectory**[a]		
	**Class 1,** Slightly increasing systolic and slightly decreasing diastolic	**Class 2,** Increasing-to-decreasing blood pressure	**Class 3,** Increasing blood pressure
**Class 1,** Slightly increasing systolic and slightly decreasing diastolic	0.862	0.035	0.103
**Class 2,** Increasing-to-decreasing blood pressure	0.068	0.823	0.109
**Class 3,** Increasing blood pressure	0.144	0.098	0.758

[a] *Estimated with multivariate LCMM*

Table B5. Posterior classification table for the three classes of blood lipids trajectories

	**Mean of the posterior probabilities of belonging to each class of blood lipids trajectory**[a]	
	**Class 1,** Slightly increasing blood lipids	**Class 2,** Increasing-to-decreasing blood lipids
**Class 1,** Slightly increasing blood lipids	0.954	0.046
**Class 2,** Increasing-to-decreasing blood lipids	0.147	0.853

[a] *Estimated with multivariate LCMM*

Tables B1-B5 suggest excellent classification for BMI, and blood lipids. The performance was poorer for the other models but still good.

Relevance of the classes

Finally, we inspected each 1-5 class models for each risk factor considered by plotting the average trajectory in each class. This allowed us to visually inspect the trajectories and ensure they were meaningful (i.e. that they were visibly distinct from each other and consistent with respect to the observed trajectories).

References

1. Nguefack HLN, Pagé MG, Katz J, et al. Trajectory modelling techniques useful to epidemiological research: A comparative narrative review of approaches. *Clin Epidemiol* 2020; **12**: 1205.
2. Proust C, Jacqmin-Gadda H, Taylor JM, Ganiayre J, Commenges D. A nonlinear model with latent process for cognitive evolution using multivariate longitudinal data. *Biometrics* 2006; **62**(4): 1014-24.
3. Proust-Lima C, Amieva H, Jacqmin-Gadda H. Analysis of multivariate mixed longitudinal data: a flexible latent process approach. *Br J Math Stat Psychol* 2013; **66**(3): 470-87.
4. Proust-Lima C, Philipps V, Diakite A, Liquet B, Proust MC. Package 'lcmm'. *CRAN R* 2023.
5. Muthén B, Muthén LK. Integrating person-centered and variable-centered analyses: growth mixture modeling with latent trajectory classes. *Alcohol Clin Exp Res* 2000; **24**(6): 882-91.
6. Morgan GB, Hodge KJ, Baggett AR. Latent profile analysis with nonnormal mixtures: A Monte Carlo examination of model selection using fit indices. *Computational Statistics & Data Analysis* 2016; **93**: 146-61.
7. Peel D, MacLahlan G. Finite mixture models. *John & Sons* 2000.
8. Lergenmuller S, Rueegg CS, Perrier F, et al. Lifetime Sunburn Trajectories and Associated Risks of Cutaneous Melanoma and Squamous Cell Carcinoma Among a Cohort of Norwegian Women. *JAMA Dermatol* 2022; **158**(12): 1367-77.
9. van de Schoot R, Sijbrandij M, Winter SD, Depaoli S, Vermunt JK. The GRoLTS-Checklist: Guidelines for Reporting on Latent Trajectory Studies. *Structural Equation Modeling: A Multidisciplinary Journal* 2017; **24**(3): 451-67.

Supplementary Methods 3: Overview of sensitivity analyses

We conducted sensitivity analyses to evaluate the effect of the various choices made in the study. The study already includes a high number of supplements, we therefore only briefly describe the analyses here. Additional information may be provided upon reasonable request.

Sex-adjusted latent class analysis (LCA) models

The LCA models allow for the inclusion of covariates. However, in this study, for simplicity and to avoid interpretability issues, we used the "unconditional" LCA model, without covariates. Nonetheless, to evaluate the influence of sex on the class identification, we also tested sex-adjusted LCA models (in which sex can influence the clusters) and sex-stratified LCA models a model (unconditional models for women and men separately). These yielded similar clusters as those presented in this study.

Latent class analysis models including harmful use of alcohol

We only had information on all NCD risk factors including harmful use of alcohol for 78,941 individuals. The high percentage of missing is mainly due to the earlier studies (1970s-1980s) not asking questions about alcohol consumption. Because this is a systematic lack of information, and because of the high percentage of missing, we did not present LCA results including harmful use of alcohol. Nonetheless, we did conduct such analyses. The models identified the same clusters as presented in this study, but instead of dyslipidaemia, identified a cluster of individuals with harmful use of alcohol that were also smokers.

Latent class mixed models (LCMMs) for harmful use of alcohol.

For the same reasons mentioned above, we chose not to present LCMMs for mean alcohol consumption (grams/14 days). In total, 11,506 individuals had at least one observation for mean alcohol consumption in the time window considered, for a total of 26,263 observations. The model identified 4 classes of mean alcohol consumption trajectories (stable low, low to high, high to low, high to very high), with a performance similar to the LCMMs for the other risk factors.

Alternative timescale for the latent class mixed models

For the LCMMs, we used age as timescale, and restricted the sample to those with at least 3 participations in the time window 30–70 years, whereof one in the age span 30–40 years (trajectory entry) and one in the age span 60–70 years. This reduced the sample size considerably (n=22,428). There are several ways in which these restrictions can be weakened.

Shortening the window and identifying trajectories from 40–70 years or 40–60 years slightly increases the sample size (to about 29,000). Using these windows, and somewhat expectedly, we identify trajectories that are "truncated" compared to the trajectories presented in this study.

Allowing less than 3 participations in the time window increases the sample size but puts more weight on trajectories without curvature. This resulted in more participants placed in the linear (stable) trajectories and/or participants allocated to trajectories with a lower posterior probability.

Using time as the timescale instead of age considerably increases the sample size, because it only imposes restrictions on time, not age. We estimated trajectories using time as timescale for smoking intensity, leisure-time physical activity, and body mass index. This increases the sample size to about 90,000 and yields very similar trajectories to those presented in the paper. However, age is an important factor for both lifestyle and biological risk factors. We therefore used age as timescale in the study.

Alternative models for the latent class mixed models

There are several things to consider when building an LCMM (see Supplementary Methods 2), which rarely leads to only one possible model. Other model specifications included:

- Models with covariance structures instead of random intercepts
- Models including a random effect of age
- Alterative transformations of the risk factors (eg. log-transformation, different splines specifications)
- Age modeled as third order polynomial

These all lead to very similar results, but usually took longer time to run.
