# Supplementary material for: Clustering and trajectories of key noncommunicable disease risk factors in Norway: the NCDNOR project
Source: Sci Rep. 2023 Sep 2;13:14479. doi: 10.1038/s41598-023-41660-x (PMC10475033; doi:10.1038/s41598-023-41660-x)
Supplement: Supplementary file 3 — Supplementary Tables. [file 41598_2023_41660_MOESM3_ESM.pdf]

Supplementary Tables

Knut Eirik Dalene, Simon Lergenmuller et al. Clustering and trajectories of key noncommunicable disease risk factors in Norway – the NCDNOR project

Supplementary Table S1. A detailed description of the included health studies 2

Supplementary Tables S2. 5

Supplementary Table S2a. Overview of variables used to generate the harmonized variables smoking status, cigarettes/day, and smoking years 5

Supplementary Table S2b. Harmonization and data cleaning 7

Supplementary Tables S3. 8

Supplementary Table S3a. Overview of the questionnaire items (English version) used to assess leisure-time physical activity (LTPA) in the included health studies 8

Supplementary Table S3b. Description of the adapted Norwegian version of the Saltin-Grimby Physical Activity Scale used to assess leisure-time physical activity in several of the included studies [in Norwegian] 10

Supplementary Table S3c. Description and of 'the CONOR instrument' used to assess leisure-time physical activity in CONOR, HUNT2, HUNT3, the third and fourth round of data collection in the Age 40 Program, Tromsø4, And Tromsø5 14

Supplementary Table S3d. Description and of 'the HUNT instrument' used to assess leisure-time physical activity in HUNT1, HUNT3, HUNT4, HUNT4ST, Tromsø6, and Tromsø7 15

Supplementary Table S3e. Harmonization of leisure-time physical activity (LTPA) assessed using the Saltin-Grimby Physical Activity Level Scale (SGPALS) and 'the CONOR instrument' into the SGPALS 16

Supplementary Table S3f. Harmonization of leisure-time physical activity assessed by the Saltin-Grimby Physical Activity Level Scale (SGPALS) and 'the HUNT instrument' into SGPALS 17

Supplementary Table S4. Overview of the questionnaire items used to assess alcohol intake in the included health studies 18

Supplementary Table S5. Proportion of missing information in the included health studies by health study participation 19

Supplementary Table S6. Participant characteristics at participation 1, 2, and 3 among individuals with at least 3 participations (n=110,512) 21

Supplementary Table S7. Participant characteristics by cluster of noncommunicable disease risk factors (n=625,364) 22

Supplementary Table S8. Participant characteristics at trajectory entry by latent class of smoking intensity trajectory (n=22,412) 24

Supplementary Table S9. Participant characteristics at trajectory entry by latent class of leisure-time physical activity trajectory (n=22,425) 25

Supplementary Table S10. Participant characteristics at trajectory entry by latent class of body mass index trajectory (n=22,391) 26

Supplementary Table S11. Participant characteristics at trajectory entry by latent class of blood pressure trajectory (n=22,403) 27

Supplementary Table S12. Participant characteristics at trajectory entry by latent class of blood lipids trajectory (n=22,400) 28

Supplementary Table S1. A detailed description of the included health studies

Study	Description
The Norwegian Counties Study	The study included participants in three (out of 19 in total) Norwegian counties with a mainly rural settlement (Finnmark, Sogn og Fjordane, and Oppland). Originally, three rounds of data collection were conducted in the 1970s and 80s. These three first studies included anthropometric measurements, blood pressure measurements, non-fasting blood samples and a questionnaire.<br>In Finnmark, the 1st study was carried out in 1974–75, in Sogn og Fjordane in 1975–76, and in Oppland in 1976–78. For the first study, all residents aged 35–49 years were invited to participate. In addition, a 10% random sample of residents aged 20–34 was invited in Sogn og Fjordane and Oppland. In Finnmark, all residents aged 20–34 years were invited in four of the county's municipalities in addition to a 10% random sample in the remaining municipalities. The overall participation in the 1st study was 88%.[1]<br>The 2nd study was carried out after three years in Finnmark (1978–79), and after five years in Sogn og Fjordane (1980–81) and Oppland (1981–83). The overall participation in the 2nd study was 88%.[2] The following were invited to the 2nd study:<br>• Finnmark<br>o All residents aged 35–52 on December 31st 1977 (born 1925–1942)<br>o Everyone invited to the 1st study still residing in Finnmark aged 23–34<br>o An 11% random sample of residents not invited to the 1st study aged 23–34<br>o A 10% random sample of residents aged 20–22<br>• Sogn og Fjordane<br>o All residents aged 40–54 on December 31st 1980 (born 1926–1940)<br>o Everyone invited to the 10% random sample in the 1st study still residing in Sogn og Fjordane aged 25–39<br>o An 11% random sample of residents not invited to the 1st study aged 25–39<br>o A 10% random sample of residents aged 20–24<br>o A 50% random sample of residents aged 17–19 in three of the county's municipalities<br>• Oppland<br>o All residents aged 40–54 on December 31st 1981 (born 1927–1941)<br>o Everyone invited to the 10% random sample in the 1st study still residing in Oppland aged 25–39<br>o A 5% random sample of residents aged 20–24<br>Minor modifications were made before the 3rd study, which was carried out 1987–1988 in Finnmark, 1985–1986 in Sogn og Fjordane, and 1986–1988 in Oppland, but the main core of participants in the previous rounds were invited. This resulted in a sample of residents aged 20–49 years comparable to the samples aged 20–49 years in the 1st and 2nd study, and a prospective sample with two or three participations (aged from 20 years at the first participation to 63 years at third participation). For the cohort aged 45–49 years at the 1st study, capacity constraints limited invitations to a 10% random sample.[3] The overall participation in the 3rd study was 84%.<br>In 2006–2008, a follow-up questionnaire was sent to all those still alive that had participated at least once previously and were born between 1925–1947 (Finnmark), 1926–1945 (Sogn og Fjordane) and 1927–1946 (Oppland) (i.e. aged 59–83 years. The overall participation in this 4th study was 59%. The 4th study was questionnaire based only.<br>In NCDNOR, 94,022 participants of the Norwegian Counties Study were included (Figure 1). We excluded participants aged <20 years (n=454), resulting in 93,568 individuals born 1925–1967 (Figure 2), >50,000 of which participated ≥3 times.
The Age 40 Program Oslo	The study was originally organized as a cardiovascular disease (CVD) risk screening by the Oslo municipality health council in collaboration with the National Health Screening Service in Norway [4] The screening included anthropometric measurements, blood pressure measurements, non-fasting blood samples and a questionnaire, with all Oslo residents invited between 1981 and 1988 the year they turned 40 years of age (participation 55%).[5] A similar, restructured screening did continue in some districts of Oslo until 1999, but the quality of the data collected after 1988 was reported to have poor quality.[6]

In total (1981–1999), 20,740 women and 18,754 men (from 104,482 invited) participated in the Age 40 Program Oslo.[7] NCDNOR includes 37,155 participants of the Age 40 Program Oslo (Figure 1). We excluded participants with unreliable age or inclusion date (n=69), and participants included after 1988 (n=13,200), resulting in 23,886 individuals born 1934–1949 (Figure 2).

The Age 40 Program

Inspired by the Age 40 Program in Oslo, the National Health Screening Service in Norway initiated cardiovascular disease risk screening programs in several other counties between 1985 and 1987, which also including anthropometric measurements, blood pressure measurements, non-fasting blood samples and a questionnaire, and by 1993 all Norwegian counties had conducted at least one screening.[8] All individuals residing in the county and aged 40–42 years by December 31st the year the study started in that county were invited.[9] Some counties also invited residents aged <40 and 43–44 years, and between 1985 and 1993, three counties also invited those aged 65–67 years.[10] Some individuals moved between counties and were invited more than once (exact number unknown), resulting in ~10,000 individuals participating several times. The overall participation was ~69%, with higher rates in the beginning (e.g. Østfold county: 81% in 1985 vs. 52% in 1999).[11]

The measurement protocols of the Age 40 Program remained relatively unchanged over time and was almost identical to the one used in the Norwegian Counties Study,[8] but the questionnaire was revised three times (1988, 1994, 1997) and grew from one to three pages. Some counties/municipalities included additional measurements and/or questionnaire items some of the years the study was conducted.[7]

NCDNOR includes 417,097 participants of the Age 40 Program (Figure 1). We excluded participants aged <20 years (n=153), resulting in 416,944 individuals born 1900–1974 (Figure 2).

The HUNT Study

The HUNT Study is the largest running health study in Norway and has been conducted four times (1984–1986, 1995–1997, 2008, and 2018–2019 (Figure 1)). It is designed to cover a broad range of health-related topics via questionnaires, interviews, clinical examinations, laboratory measurements and storage of biological samples. Following the completion of the fourth wave of the HUNT Study, which for the first time included participants from both the (former) North- and South-Trøndelag counties, an updated cohort profile for the HUNT Study was published giving detailed descriptions and information on the different studys.[12]

The participation ranges from 89% in 1984–86 to 43% in 2018–19 (HUNT4 in South-Trøndelag (HUNT4ST)).[12] Almost 250,000 individuals have participated in HUNT, totalling 380,000 person-observations (~40,000 having participated three or four times). NCDNOR includes 228,955 participants from the HUNT Study. We excluded participants aged <20 years (n=3,039) resulting in 225,916 individuals born 1882–1999 (Figure 2). More details about the HUNT study are provided through the HUNT research centre's web pages (https://www.ntnu.edu/hunt) and the HUNT databank (https://hunt-db.medisin.ntnu.no/hunt-db/)

The Tromsø Study

The Tromsø Study is the longest running health study in Norway, including seven studies (Tromsø1–Tromsø7: 1974, 1979–1980, 1986–1987, 1994–1995, 2001, 2007–2008, and 2015–2016). The study has evolved from a combined cardiovascular disease risk screening and research study in the 1970s to a multipurpose health study covering a broad range of topics with data collections including questionnaires, interviews, biological sampling, and clinical examinations. An updated cohort profile was published recently giving detailed descriptions and information on the different Tromsø studies.[13] Participation ranges from 79% in Tromsø5 to 65% in Tromsø7. NCDNOR includes 36,648 participants of Tromsø4–Tromsø7, born 1897–1977 (Figures 1–2), of which >30,000 participated more than once. More details about the Tromsø study are provided through the study's web pages (https://uit.no/research/tromsostudy) and databank (https://helsedata.no/no/variabler/?datakilde=K_TR&page=search)

Cohort of Norway (CONOR)

Created as a collaborative project between epidemiological research centres in Norway, the 'Cohort of Norway' (CONOR) includes data on participants from 10 regional epidemiological studies conducted between 1994 and 2003 (Tromsø4–5; HUNT2; 'HUSK' (The Hordaland Health Study); 'Oslo II' (The second Oslo Study); 'HUBRO' (The Oslo Health Study); 'I-HUBRO' (The Oslo Immigrant Health Study); 'OPPHED' (The Oppland and Hedmark Health Study); 'TROFINN' (The Troms and Finnmark Health Study), and; 'MoRo II' (The second part of the Romsås in Motion Study).[14] The studies used the same protocols for anthropometric measurements, blood pressure measurements and non-fasting blood, and all studies used about 50 core CONOR questions agreed upon before the first CONOR study in Tromsø in 1994.[15,16] Most of the individual studies also made several additional measurements and included additional questionnaire items.[16] The overall participation rate in CONOR was ~58% but varied between studies from ~30% (I-HUBRO) to

~76% (Tromsø 5). NCDNOR includes 173,173 CONOR participants.[15] We excluded participants of the HUNT Study and the Tromsø Study (n=92,643), and participants aged <20 years (n=23), resulting in 80,507 participants born 1920–1982 (Figure 2). More details about CONOR can be found elsewhere.[14-17]

References

1. Bjartveit, K., Foss, O. P., Gjervig, T. & Lund-Larsen, P. G. The cardiovascular disease study in Norwegian counties. Background and organization. *Acta medica Scandinavica Supplementum* 1979; **634**: 1-70.
2. The National Health Screening Service. The Cardiovascular disease study in Norwegian counties - Results from the second screening. 15-16, 1988.
3. Tverdal, A. & Bjartveit, K. Health consequences of reduced daily cigarette consumption. *Tobacco control* 2007; **15**: 472-80.
4. Jenum, A. K., Stensvold, I., Bjartveit, K., Thelle, D. S. & Hjermann, I. Risikofaktorer for hjerte- og karsykdom i Oslo, Sogn og Fjordane og Finnmark. *Tidsskrift for Den Norske Laegeforening* 1998.
5. Jenum, A. K., Graff-Iversen, S., Selmer, R. & Søgaard, A.-J. Risikofaktorer for hjerte-og karsykdom og diabetes gjennom 30 år. *Tidsskrift for Den norske legeforening* 2007.
6. Holme, I., Tonstad, S. & Hjermann, I. Risikofaktorer for hjerte- og karsykdom blant 40-åringer i Oslo 1981 – 99. *Tidsskrift for Den Norske Laegeforening* 2004.
7. Folkehelseinstituttet. 40-åringsundersøkelsene 1985-1999 2015. https://www.fhi.no/studier/helseundersokelser/helseundersokelser/40-aringsundersokelsene/ (accessed January).
8. Bjartveit, K. Statens helseundersøkelser: Fra tuberkulosekamp til mangesidig epidemiologisk virksomhet. *Norsk Epidemiologi* 2009; **7**(2).
9. Wøien, G., Øyen, O. & Graff-Iversen, S. 22 år med hjerte- og karundersøkelser i norske fylker. Bør vi være tilfreds med den utviklingen risikofaktorene har hatt? *Norsk Epidemiologi* 2009; **7**(2).
10. Norwegian Institute of Public Health Landsomfattende helseundersøkelser (LHU). https://www.fhi.no/div/helseundersokelser/landsomfattende-helseundersokelser-lhu/ (accessed 13.01.2023).
11. Tverdal, A. & Selmer, R. M. 40-åringsundersøkelsene–400 000 menn og kvinner har møtt opp. *Tidsskrift for Den norske legeforening* 2002.
12. Åsvold, B. O. *et al.* Cohort Profile Update: The HUNT Study, Norway. *International journal of epidemiology* 2022.
13. Hopstock, L. A. *et al.* The seventh survey of the Tromsø Study (Tromsø7) 2015-2016: study design, data collection, attendance, and prevalence of risk factors and disease in a multipurpose population-based health survey. *Scandinavian journal of public health* 2022: 14034948221092294.
14. Engeland, A. & Søgaard, A. CONOR (COhort NORway) - en oversikt over en unik forskningsdatabank. *Norsk Epidemiologi* 2009; **13**.
15. Naess, O. *et al.* Cohort profile: cohort of Norway (CONOR). *International journal of epidemiology* 2008; **37**(3): 481-5.
16. Norwegian Institute of Public Health Cohort of Norway (CONOR). https://www.fhi.no/studier/cohort-of-norway/ (accessed 16.01.2023).
17. Aamodt, G., Søgaard, A. J., Naess, Ø., Beckstrøm, A. C. & Samuelsen, S. O. [The CONOR database--a little piece of Norway]. *Tidsskrift for den Norske laegeforening : tidsskrift for praktisk medicin, ny raekke* 2010; **130**(3): 264-5.

Supplementary Tables S2

Supplementary Table S2a. Overview of variables used to generate the harmonized variables smoking status, cigarettes/day, and smoking years

Study:	CONOR	HUNT					NCS				A40P				A40PO	TROMSØ			
Cohort:	1	1	2	3	4	4S	1	2	3	4	1	2	3	4	1	4	5	6	7
**Harmonized variables:**																			
Smoking status (Never, Former, Current (daily smoker))	x	x	x	x	x	x	x	x	x	x	x	x	x	x	x	x	x	x	x
Cigarettes/day (current or former daily smoker)	x	x	x	x	x	x	x	x	x	x	x	x	x	x	x	x	x	x	x
Smoking years (current of former daily smoker)	x	x	x	x	x	x	x	x	x	x	x	x	x	x	x	x	x	x	x
**Original variables*:**																			
**Smoking status summarized**																			
3 cat: never daily - former daily - current daily		x	x																
4 cat: never - former - current daily - sometimes daily				x															
5 cat: never - former daily - current daily - sometimes daily - sometimes former					x	x													
3 cat: not current daily - current cigarettes daily - current cigars/pipe							x	x											
2 cat: never daily cigarette smoker - not current daily but former daily cigarette smoker							x	x											
3 cat: current - former - never	x									x							x	x	x
3 cat: current daily - sometimes daily - never																	x		
**Smoking status separate variables**																			
Never smoked				x	x	x													
Never daily smoker			x											x	x				
Current daily?	x	x			x	x	x	x	x		x				x				
If yes, cigarettes daily?															x				
Cigarettes daily	x	x	x	x			x	x	x	x	x	x	x	x		x	x		
Cigars/cigarillos/pipe daily				x															
Current daily pipe	x	x	x				x	x	x		x	x	x	x		x	x		
Current daily cigars	x	x	x				x	x	x		x	x	x	x		x	x		
Former current daily					x	x						x							
Former current daily cigarettes		x					x	x	x	x	x				x				
Former smoker				x															
**Number of cigarettes**																			
I smoke approximately N cigarettes per day {if current daily smoker}					x	x	x	x		x									
For previous or current smokers: how many cigarettes daily? (min, max, mean)																x			
Number of cigarettes currently or formerly smoked daily {if current or former daily smoker}	x	x	x	x							x	x			x		x	x	x
Number of cigarettes currently or formerly smoked daily			x	x															
Number of cigarettes currently or formerly smoked daily - from							x	x	x		x	x	x	x					
Number of cigarettes currently or formerly smoked daily - to							x	x	x		x	x	x	x					
I used to smoke N cigarettes per day {If former daily smoker}					x	x				x									
**Years smoking/Age when starting**																			

Total number of years of daily smoking		x					x	x	x		x	x	x	x		x	x	x	x
Total number of years of daily smoking2		x	x	x															
I started smoking daily when I was __ years old {if current daily smoker}					x	x				x									
I started smoking daily when I was __ years old {if current or former daily smoker}		x	x	x												x	x	x	x
I started smoking daily when I was __ years old {if former daily smoker}					x	x				x									
I started smoking when I was __ years old {if current smoker}	x												x	x					
No of years smoking	x														x				
**Years since cessation/Age when quitting**																			
I was __ years old when I stopped smoking {if current former daily smoker}				x															
I was __ years old when I stopped smoking {if former daily smoker}					x	x				x									

Abbreviations: CONOR, Cohort of Norway; HUNT, the Trøndelag Health Study (4S refers to the HUNT4 in South-Trøndelag); NCS, the Norwegian Counties Study; A40P, the Age 40 Program; A40PO, the Age 40 Program Oslo.

* Translated from Norwegian to English

Supplementary Table S2b. Harmonization and data cleaning*

Variable	
Smoking status	Harmonized into variable with three categories (**0 Never, 1 Former daily smoker, 2 Current daily smoker**) using a combination of original summary variables (‘Smoking status summarized’) and separate dichotomous variables (‘Smoking status separate variables’). The syntaxes (Stata do-files) used to harmonize the data is available upon request to the corresponding author.
Cigarettes/day	Harmonized from original variables, which for all surveys except the Age 40 Program Oslo were numerical in their original form. In the Age 40 Program Oslo, the original variable was categorical (0, 1–4, 5–9, 10–14, 15–19, 20–24 and ≥25 cigarettes per day). For the present study we harmonized cigarettes per day from the Age 40 Program Oslo with data from the other surveys by creating a numerical variable from these categories using the following rules: 0=*0*, 1–4=*2.5*, 5–9=*7*, 10–14=*12*, 15–19=*17*, 20–24=*22* and ≥25=*27* cigarettes per day. The syntaxes (Stata do-files) used to harmonize the data is available upon request to the corresponding author.
Smoking years	Except for (HUNT4, HUNT4ST, the 4th Norwegian Counites study), *Smoking years* was harmonized using the original numerical variables. For HUNT4, HUNT4ST, the 4th Norwegian Counites study we calculated *Smoking years* from self-reported age of daily smoking initiation and cessation (or age at participation if current daily smoker). The syntaxes (Stata do-files) used to harmonize the data is available upon request to the corresponding author.

*We made the following data cleaning decisions:

1) cigarettes/day and smoking years set to 0 if smoking status = never

2) cigarettes/day set to missing if >100

3) smoking years set to missing if >90.

Supplementary Tables S3

Supplementary Table S3a. Overview of the questionnaire items (English version) used to assess leisure-time physical activity (LTPA) in the included health studies

Study:	CONOR	HUNT					NCS				A40P				A40PO	Tromsø			
Cohort:	1	1	2	3	4	4S	1	2	3	4	1	2	3	4	1	4	5	6	7
**Leisure-time physical activity /exercise harmonized**	x	x	x	x	x	x	x	x	x	x	x	x	x	x	x	x	x	x	x
**Saltin-Grimby physical activity scale (SGPALS) – see next tables**																			
1 Almost completely inactive: reading, TV watching, movies, etc.<br>2 Some physical activity during at least 4 hours per week: riding a bicycle or walking to work, walking, or skiing with the family, gardening.<br>3 Regular activity: such as heavy gardening, running, calisthenics, tennis, etc.<br>4 Regular hard physical training for competition in running events, soccer, racing, European handball, etc. several times per week.	x						x	x	x	x	x	x		x	x		x	x	x
**'CONOR questions' – see next tables**																			
Number of hours with hard physical activity per week past year<br>(4 cat: none, <1h, 1–2h, 3h+)	x		x	x									x	x		x	x		
Number of hours with light physical activity per week past year<br>(4 cat: none, <1h, 1–2h, 3h+)	x		x	x									x	x		x	x		
**'HUNT questions' – see next tables**																			
How often do you exercise?<br>(5 cat: never, <1/week, 1/week, 2–3/week, 4+/week)		x		x	x	x												x	x
How hard do you exercise if you exercice 1+/week<br>(3 cat: take it easy without getting out of breath or sweating, hard enough to be out of breath and sweating, almost to exhaustion)		x		x	x	x												x	x
How long do you exercise if you exercise 1+/week<br>(4 cat: <15mn, 15–19mn, 30–60mn, >60mn)		x		x	x	x												x	x
**Other questions (not used in the present study)**																			
Have at least 30 min physical activity at work and/or in leisure time<br>(yes/no)				x															
Number of times training or doing sports last 6 months<br>(5 cat: >1/week, 1/week, 1–3/month, 1–5 last 6 month, never)				x	x	x													
Occupational physical activity (SGPALS)																			
1 You are mainly sedentary and do not walk much around at your workplace, for example, desk work, work including assembling of minor parts.<br>2 You walk around quite a bit at your workplace but do not have to carry heavy items, for example, light industrial work, non-sedentary office work, inspection and the like.	x			x			x	x	x	x					x	x	x	x	x

3 Most of the time you walk, and you often have to walk up stairs and lift various items. Examples include mail delivery and construction work.
4 You have heavy physical work. You carry heavy burdens and carry out physically strenuous work, for example, work including digging and shovelling

Abbreviations: CONOR, Cohort of Norway; HUNT, the Trøndelag Health Study (4S refers to the HUNT4 in South-Trøndelag); NCS, the Norwegian Counties Study; A40P, the Age 40 Program; A40PO, the Age 40 Program Oslo.

Supplementary Table S3b. Description of the adapted Norwegian version of the Saltin-Grimby Physical Activity Scale* used to assess leisure-time physical activity in several of the included studies [in Norwegian]. Deleted and added text as compared to the earliest studies (the Norwegian Counties Study (1974–1988)) highlighted in **bold**.

**Survey**	
The Nor. Count. study 1 (1974–78)	Bevegelse og kroppslig anstrengelse i deres **fritid**. Hvis aktiviteten varierer meget f.eks. mellom sommer og vinter så ta et gjennomsnitt. Spørsmålet gjelder bare det siste året. Sett kryss i ruten hvor 'JA' passer best.<br>1. Leser, ser på fjernsyn eller annen stillesittende beskjeftigelse?<br>2. Spaserer, sykler eller beveger Dem på annen måte minst **4 timer i uken**?<br>(Heri medregnes også gang eller sykling til arbeidsstedet, søndagsturer m.m.)<br>3. Driver mosjonsidrett, tyngre hagearbeid e.l.?<br>(Merk at virksomheten skal vare **minst 4 timer** i uken)<br>4. Trener hardt eller driver konkurranseidrett, regelmessig og **flere ganger i uken**?
The Nor. Count. study 2 (1977–83)	Bevegelse og kroppslig anstrengelse i deres **fritid**. Hvis aktiviteten varierer meget f.eks. mellom sommer og vinter så ta et gjennomsnitt. Spørsmålet gjelder bare det siste året. Sett kryss i ruten hvor 'JA' passer best.<br>1. Leser, ser på fjernsyn eller annen stillesittende beskjeftigelse?<br>2. Spaserer, sykler eller beveger Dem på annen måte minst **4 timer i uken**?<br>(Heri medregnes også gang eller sykling til arbeidsstedet, søndagsturer m.m.)<br>3. Driver mosjonsidrett, tyngre hagearbeid e.l.?<br>(Merk at virksomheten skal vare **minst 4 timer** i uken)<br>4. Trener hardt eller driver konkurranseidrett, regelmessig og **flere ganger i uken**?
The Nor. Count. study 3 (1985–88)	Bevegelse og kroppslig anstrengelse i deres **fritid**. Hvis aktiviteten varierer meget f.eks. mellom sommer og vinter så ta et gjennomsnitt. Spørsmålet gjelder bare det siste året. Sett kryss i ruten hvor 'JA' passer best.<br>1. Leser, ser på fjernsyn eller annen stillesittende beskjeftigelse?<br>2. Spaserer, sykler eller beveger Dem på annen måte minst **4 timer i uken**?<br>(Heri medregnes også gang eller sykling til arbeidsstedet, søndagsturer m.m.)<br>3. Driver mosjonsidrett, tyngre hagearbeid e.l.?<br>(Merk at virksomheten skal vare **minst 4 timer** i uken)<br>4. Trener hardt eller driver konkurranseidrett, regelmessig og **flere ganger i uken**?
The Nor. Count. study 4 (2006–08)	Bevegelse og kroppslig anstrengelse i deres **fritid**. Hvis aktiviteten varierer meget f.eks. mellom sommer og vinter så ta et gjennomsnitt. **~~Spørsmålet gjelder bare det siste året.~~** **(**Sett kryss i **den** ruten **~~hvor 'JA'~~** **som** passer best**)**.<br>1. Leser, ser på fjernsyn eller annen stillesittende beskjeftigelse?<br>2. Spaserer, sykler eller beveger **~~Dem~~** **deg** på annen måte **~~minst 4~~** **2–4 timer i uken**?<br>**~~(Heri medregnes også gang eller sykling til arbeidsstedet, søndagsturer m.m.)~~**<br>3. Spaserer, sykler eller beveger **~~Dem~~** **deg** på annen måte minst **4 timer i uken**?<br>**~~(Heri medregnes også gang eller sykling til arbeidsstedet, søndagsturer m.m.)~~**<br>4. Driver mosjonsidrett, tyngre hagearbeid e.l.?<br>(Merk at **~~virksomheten~~** **aktiviteten** skal vare minst 4 timer i uken)

	5. Trener hardt eller driver konkurranseidrett, regelmessig og flere ganger i uken?
The Age 40 program Oslo (1981–1988)	Bevegelse og kroppslig anstrengelse i deres fritid. Hvis aktiviteten varierer meget f.eks. mellom sommer og vinter så ta et gjennomsnitt. Spørsmålet gjelder bare det siste året. Sett kryss i ruten hvor 'JA' passer best.<br>1. Leser, ser på fjernsyn eller annen stillesittende beskjeftigelse?<br>2. Spaserer, sykler eller beveger Dem på annen måte minst 4 timer i uken?<br>(Heri medregnes også gang eller sykling til arbeidsstedet, søndagsturer m.m.)<br>3. Driver mosjonsidrett, tyngre hagearbeid e.l.?<br>(Merk at virksomheten skal vare minst 4 timer i uken)<br>4. Trener hardt eller driver konkurranseidrett, regelmessig og flere ganger i uken?
The Age 40 program (1985–1987, 1988–1994)	Bevegelse og kroppslig anstrengelse i deres fritid. Hvis aktiviteten varierer meget f.eks. mellom sommer og vinter så ta et gjennomsnitt. Spørsmålet gjelder bare det siste året. Sett kryss i ruta hvor 'JA' passer best.<br>1. Leser, ser på fjernsyn eller annen stillesittende beskjeftigelse?<br>2. Spaserer, sykler eller beveger Dem på annen måte minst 4 timer i uken?<br>(~~**Heri medregnes også**~~ **Her skal De også regne med** gang eller sykling til arbeidsstedet, søndagsturer m.m.)<br>3. Driver mosjonsidrett, tyngre hagearbeid e.l.?<br>(Merk at ~~**virksomheten**~~ **aktiviteten** skal vare minst 4 timer i uken)<br>4. Trener hardt eller driver konkurranseidrett, regelmessig og flere ganger i uken?
The Age 40 program 1994–1997	N/A (leisure-time physical activity assessed using an instrument used in the CONOR surveys (see below))
The Age 40 program (1997–1999)	Bevegelse og kroppslig anstrengelse i deres fritid. Hvis aktiviteten varierer meget f.eks. mellom sommer og vinter så ta et gjennomsnitt. Spørsmålet gjelder bare det siste året. Sett kryss i **den** ruta ~~**hvor 'JA'**~~ **som** passer best.<br>1. Leser, ser på fjernsyn eller annen stillesittende beskjeftigelse?<br>2. Spaserer, sykler eller beveger Dem på annen måte minst 4 timer i uken?<br>(~~**Heri medregnes også**~~ **Her skal du også regne med** gang eller sykling til arbeidsstedet, søndagsturer m.m.)<br>3. Driver mosjonsidrett, tyngre hagearbeid e.l.?<br>(Merk at ~~**virksomheten**~~ **aktiviteten** skal vare minst 4 timer i uken)<br>4. Trener hardt eller driver konkurranseidrett, regelmessig og flere ganger i uken?
The Tromsø Study 4 (1994–1995)	N/A (leisure-time physical activity assessed using an instrument used in the CONOR surveys (see below))
The Tromsø Study 5 (2001)	**Angi** bevegelse og kroppslig anstrengelse i deres fritid. Hvis aktiviteten varierer meget f.eks. mellom sommer og vinter så ta et gjennomsnitt. Spørsmålet gjelder bare det siste året. **(**Sett kryss i **den** ruten ~~**hvor 'JA'**~~ **som** passer best**)**.<br>1. Leser, ser på fjernsyn eller annen stillesittende beskjeftigelse?<br>2. Spaserer, sykler eller beveger Dem på annen måte minst 4 timer i uken?<br>(~~**Heri medregnes også**~~ **Her skal De også regne med** gang eller sykling til arbeidsstedet, søndagsturer m.m.)<br>3. Driver mosjonsidrett, tyngre hagearbeid e.l.?<br>(Merk at virksomheten skal vare minst 4 timer i uken)<br>4. Trener hardt eller driver konkurranseidrett, regelmessig og flere ganger i uken?

The Tromsø Study 6 (2007–2008)	**Angi** bevegelse og kroppslig anstrengelse i deres fritid. Hvis aktiviteten varierer meget f.eks. mellom sommer og vinter så ta et gjennomsnitt. Spørsmålet gjelder bare det siste året. **(**Sett kryss i **den** ruten ~~**hvor 'JA'**~~ **som** passer best**)**.<br>1. Leser, ser på fjernsyn eller annen stillesittende beskjeftigelse?<br>2. Spaserer, sykler eller beveger Dem på annen måte minst 4 timer i uken?<br>(~~**Heri medregnes også**~~ **Her skal De også regne med** gang eller sykling til arbeidsstedet, søndagsturer m.m.)<br>3. Driver mosjonsidrett, tyngre hagearbeid, **snømåking** e.l.?<br>(Merk at ~~**virksomheten**~~ **aktiviteten** skal vare minst 4 timer i uken)<br>4. Trener hardt eller driver konkurranseidrett, regelmessig og flere ganger i uken?
The Tromsø Study 7 (2015–2016)	**Angi** bevegelse og kroppslig anstrengelse i deres fritid **det siste året**. Hvis aktiviteten varierer ~~**meget f.eks. mellom sommer og vinter så**~~ **gjennom året,** ta et gjennomsnitt. ~~**Spørsmålet gjelder bare det siste året.**~~ **(**Sett kryss i **den** ruten ~~**hvor 'JA'**~~ **som** passer best**)**.<br>1. Leser, ser på ~~**fjernsyn**~~ **TV/skjerm** eller annen stillesittende beskjeftigelse?<br>2. Spaserer, sykler eller beveger Dem på annen måte minst 4 timer i uken?<br>(~~**Heri medregnes også**~~ **inkludert** gang eller sykling til arbeidsstedet, søndagsturer m.m.)<br>3. Driver mosjonsidrett, tyngre hagearbeid, **snømåking** ~~**e.l.**~~ **etc minst 4 timer i uka**?<br>~~**(Merk at virksomheten skal vare minst 4 timer i uken)**~~<br>4. Trener hardt eller driver konkurranseidrett, regelmessig og flere ganger i uken?
The HUNT Study 1 (1984–1985)	N/A (used the HUNT questions about frequency, intensity, and duration)
The HUNT Study 2 (1995–1997)	N/A (used the HUNT questions about frequency, intensity, and duration AND the instrument used in the CONOR surveys (see below))
The HUNT Study 3 (2006–2008)	N/A (used the HUNT questions about frequency, intensity, and duration AND the instrument used in the CONOR surveys (see below))
The HUNT Study 4 (2017–2019)	N/A (used the HUNT questions about frequency, intensity, and duration)
The HUNT Study 4ST (2019)	N/A (used the HUNT questions about frequency, intensity, and duration)
CONOR (1994–2003)	**Angi** bevegelse og kroppslig anstrengelse i deres fritid. Hvis aktiviteten varierer meget f.eks. mellom sommer og vinter så ta et gjennomsnitt. Spørsmålet gjelder bare det siste året. **(**Sett kryss i **den** ruten ~~**hvor 'JA'**~~ **som** passer best**)**.<br>1. Leser, ser på fjernsyn eller annen stillesittende beskjeftigelse?<br>2. Spaserer, sykler eller beveger Dem på annen måte minst 4 timer i uken?<br>(~~**Heri medregnes også**~~ **Her skal De også regne med** gang eller sykling til arbeidsstedet, søndagsturer m.m.)<br>3. Driver mosjonsidrett, tyngre hagearbeid e.l.?<br>(Merk at virksomheten skal vare minst 4 timer i uken)<br>4. Trener hardt eller driver konkurranseidrett, regelmessig og flere ganger i uken?

*The Saltin-Grimby Physical Activity Scale (original version (English))

The first section deals with physical activity within your occupation. We have classified all occupations in four groups, from sedentary to hard manual work. Please study the following table and then match your own occupation during various periods of your life with the table by checking appropriate boxes below:

1 ('Sedentary"): You are mainly sedentary and do not walk much around at your workplace—for example, desk work, work including assembling of minor parts.

2 (‘Walking”): You walk around quite a bit at your workplace but do not have to carry heavy items—for example, light industrial work, non-sedentary office work, inspection and the like.
3 (‘Walk+lift”): Most of the time you walk, and you often have to walk up stairs and lift various items. Examples include mail delivery and construction work.
4 (‘Heavy labour”): You have heavy physical work. You carry heavy burdens and carry out physically strenuous work—for example, work including digging and shovelling
*The following section deals with your **spare-time physical activity**. The table outlines four different levels. Please read the table carefully and then check appropriate boxes below*:
1 ‘Sedentary”: Almost completely inactive: reading, TV watching, movies, etc.
2 ‘Low”: Some physical activity during at least 4 hours per week: riding a bicycle or walking to work, walking or skiing with the family, gardening.
3 ‘Moderate”: Regular activity: such as heavy gardening, running, calisthenics, tennis, etc.
4 ‘High”: Regular hard physical training for competition in running events, soccer, racing, European handball, etc. several times per week.

Supplementary Table S3c. Description and of 'the CONOR instrument' used to assess leisure-time physical activity in CONOR, HUNT2, HUNT3, the third and fourth round of data collection in the Age 40 Program, Tromsø4, And Tromsø5

Question	Description of the intensity ('light' and 'hard') of the physical activity	Answer (duration)
Hvordan har din fysiske aktivitet i fritiden vært det siste året? Tenk deg et ukentlig gjennomsnitt for året. Arbeidsvei regnes som fritid.	Lett aktivitet<br>(ikke svett/andpusten)	Timer per uke i gjennomsnitt<br>1. Ingen<br>2. Under 1<br>3. 1–2<br>4. 3 el mer
	Hard fysisk aktivitet<br>(svett/andpusten)	Timer per uke i gjennomsnitt<br>1. Ingen<br>2. Under 1<br>3. 1–2<br>4. 3 el mer
**English translation:** During the last year, how has your physical activity level been during leisure-time? Think of a weekly average. Commuting to/from work is included in leisure-time.	Light physical activity<br>(not sweating/not out of breath)	Average weekly hours:<br>1. None<br>2. Less than 1 hour<br>3. 1–2 hours<br>4. 3 or more hours
	Hard physical activity<br>(sweating/out of breath)	Average weekly hours:<br>1. None<br>2. Less than 1 hour<br>3. 1–2 hours<br>4. 3 or more hours

Supplementary Table S3d. Description and of 'the HUNT instrument' used to assess leisure-time physical activity in HUNT1, HUNT3, HUNT4, HUNT4ST, Tromsø6, and Tromsø7

Introductory text	Answer (Exercise frequency):	Answer (Exercise intensity)*	Answer (Exercise duration)*
Norwegian:	Hvor ofte driver du mosjon?**<br>(Ta et gjennomsnitt)	Hvor hardt mosjonerer du?**	Hvor lenge holder du på hver gang?**
HUNT1/3, Tromsø6/7: Med mosjon mener vi at du f.eks. går tur, går på ski, svømmer eller driver trening/idrett.<br>HUNT4: Med mosjon mener vi at du f.eks. går tur, går på ski, sykler, svømmer eller driver trening/idrett.	1.Aldri<br>2. Sjeldnere enn én gang i uka<br>3. Én gang i uka<br>4. 2–3 ganger i uka<br>5. Omtrent hver dag	1.Tar det rolig uten å bli andpusten eller svett<br>2. Tar det så hardt at jeg blir andpusten og svett<br>3.Tar meg nesten helt ut	1.Mindre enn 15 minutter<br>2. 16–30 minutter<br>3. 30 minutter–1 time<br>Mer enn 1 time
English translation:	How often do you exercise?**<br>(Take an average)	How hard do you exercise?**	How long do you exercise each time?**
HUNT1/3: With exercise we mean walking, skiing, swimming, or doing exercise training/sports<br>HUNT4: With exercise we mean walking, skiing, cycling, swimming, or doing exercise training/sports	1.Never<br>2. Less than once a week<br>3. Once a week<br>4. 2–3 times a week<br>5.Nearly every day	1.I take it easy, I don't get out of breath or break into a sweat<br>2. I push myself until I'm out of breath and break into a sweat<br>3. I practically exhaust myself	1.Less than 15 minutes<br>2. 16–30 minutes<br>3. 30 minutes – 1 hour<br>More than 1 hour

*Answered if Exercise frequency = 3, 4 or 5. **Participants could only choose one category.

Supplementary Table S3e. Harmonization* of leisure-time physical activity (LTPA) assessed using the Saltin-Grimby Physical Activity Level Scale (SGPALS) and 'the CONOR instrument' into the SGPALS

Saltin-Grimby Physical Activity Level Scale levels**			The CONOR instrument**
1	('Sedentary')	←→	Light PA = 1, 2 or 3 & Hard PA = not answered
1	('Sedentary')	←→	Hard PA = 1 or 2 & Light PA = not answered
1	('Sedentary')	←→	Light PA = 1 or 2 & Hard PA = 1 or 2
1	('Sedentary')	←→	Light PA = 3 & Hard PA = 1
2	('Low')	←→	Light PA = 4 & Hard FA = not answered
2	('Low')	←→	Hard PA = 3 & Light PA = not answered
2	('Low')	←→	Light PA = 1 or 2 & Hard PA = 3
2	('Low')	←→	Light PA = 3 or 4 & Hard PA = 1 or 2
3	('Moderate')	←→	Light PA = 4 & Hard PA = 2 or 3
3	('Moderate')	←→	Light PA = 3 & Hard PA = 3
4	('High')	←→	Hard PA = 4 & Light PA = not answered
4	('High')	←→	Light PA = 1, 2, 3 or 4 & Hard PA = 4

*Harmonization decision rules were guided by four questionnaires including both SGPALS and 'the CONOR instruments' (n=114,218) which allowed cross-tabulations of original and generated SGPALS categorizations.

**Graff-Iversen S et. al. Two short questionnaires on leisure-time physical activity compared with serum lipids, anthropometric measurements and aerobic power in a suburban population from Oslo, Norway. European journal of epidemiology 2008; 23(3): 167-74.

Supplementary Table S3f. Harmonization*# of leisure-time physical activity assessed by the Saltin-Grimby Physical Activity Level Scale (SGPALS) and 'the HUNT instrument' into SGPALS

Saltin-Grimby Physical Activity Level Scale levels			The HUNT instrument
1	('Sedentary')	←→	Exercise frequency = 1 OR 2 **OR**
1	('Sedentary')	←→	'Exercise volume' at moderate intensity <0.5 hours/week (<30 minutes/week) **OR**
1	('Sedentary')	←→	'Exercise volume' at vigorous intensity <0.25 hours/week (<15 minutes/week) **OR**
1	('Sedentary')	←→	'Exercise volume' at very vigorous intensity <0.17 hours/week (<10 minutes/week)
		←→	
2	('Low')	←→	'Exercise volume' at moderate intensity 0.5-3 hours/week (30-180 minutes/week) **OR**
2	('Low')	←→	'Exercise volume' at vigorous intensity 0.25-2 hours/week (15-120 minutes/week) **OR**
2	('Low')	←→	'Exercise volume' at very vigorous intensity 0.17-0.66 hours/week (10-40 minutes/week)
		←→	
3	('Moderate')	←→	'Exercise volume' at moderate intensity ≥3 hours/week (≥180 minutes/week) **OR**
3	('Moderate')	←→	'Exercise volume' at vigorous intensity 2-5 hours/week (120-300 minutes/week) **OR**
3	('Moderate')	←→	'Exercise volume' at very vigorous intensity 0.66-3.33 hours/week (40-200 minutes/week)
		←→	
4	('High')	←→	'Exercise volume' at vigorous intensity >5 hours/week (>300 minutes/week) **OR**
4	('High')	←→	'Exercise volume' at very vigorous intensity >3.33 hours/week (>200 minutes/week)

*Prior to harmonization, the following steps, assumptions and discissions were made:

1. *Exercise frequency* categories 3-5 were re-coded on the numerical scale as follows: 3=1, 2=2.5 and 3=7 times per week. Categories 1 and 2 set to missing**.
2. *Exercise duration* categories 1-4 were re-coded on the numerical scale as follows: 1=7.5, 2=22, 3=45 and 4=90 minutes per exercise session.
3. The new *Exercise frequency* and *Exercise duration* variables were combined into a new 'Hours of exercise per week'-variable (Exercise frequency x Exercise duration)/60
4. Three new 'Exercise volume' variables were created by combining the 'Hours of exercise per week' with *Exercise intensity* assuming intensity categories 1, 2 and 3 were roughly equivalent to 'moderate intensity' (3-6 METs), 'vigorous intensity' (6-9 METs) and 'very vigorous intensity' (>9 METs), respectively.

**As per protocol (participants indicating exercise less than once a week were instructed not to answer questions about duration and intensity)

Harmonization decision rules were guided by two questionnaires including both SGPALS and 'the HUNT instrument' (n=31,128) which allowed cross-tabulations of original and generated SGPALS categorizations.

Supplementary Table S4. Overview of the questionnaire items used to assess alcohol intake in the included health studies[a]

STUDY*:	CONOR	HUNT					NCS		A40P		TROMSØ			
COHORT**:	1	1	2	3	4	4S	3	4	3	4	4	5	6	7
**Alcohol consumption yes/no**														
Are you a teetotaller? (Yes/No)	x		x				x		x	x	x	x		
Do you drink alcohol? (Yes/No)					x		x							
**Frequency of alcohol consumption categorized**														
(…) last year 1 (5 cat: several times/week, 1/week, 2-3/month, 1/month, never/seldom)	x													
(…) last year 2 (9 cat: 4-7/week, 2-3/week, 1/week, 2-3/month, 1/month, <12/year, not last year, never, (teetotaller))	x			x				x				x		
(…) last year 3 (6 cat: not the last year, max 1/month, 2-4/month, 2-3/week, 4+/week, never drinks)					x	x								
(…) last year 4 (5 cat: not last year, a few times, 1-3/month, 1-2/week, >2/week)							x							
(…) last year 5 (6 cat: No, never, <1/month, monthly, weekly, daily/almost daily)														x
(…) now (5 cat: Never, Monthly, or less, 2-4/month, 2-3/week, 4+/week)													x	x
(…) last 2 weeks (5 cat: none but not a teetotaller, 1-4 times, 5-10 times, >10 times, I never drink/teetotaller)		x												
(…) beer/wine/spirits last week (4 cat: not, 1 time, 2-3 times, 4 or more)							x							
(…) beer/wine/spirits consumption now (5 cat: never/few times a year, 1-2/month, 1/week, 2-3/week, every day)							x				x			
**Frequency of alcohol consumption numerical**														
Number of times/days per month drinking alcohol (≥9 times equivalent to ≥2 times/week)	x		x						x	x	x	x		
**Number of units/glasses/bottles/drinks/grams drunken**														
Number of units beer/wine/spirits per 2 weeks usually	x		x	x	x	x			x	x	x	x		
**Heavy drinking – How often … on the same occasion**														
(…) do you drink ≥5 units of alcohol on the same occasion? (4 cat: never, monthly, weekly, daily)				x										
(…) do you drink ≥5 units of alcohol (…)? (5 cat: not the past year, a few times, 1-2/month, 1-2/week, 3+/week)											x			
(…) do you drink ≥6 units of alcohol (…)? (5 cat: never, less <1/month, monthly, weekly, daily or almost daily)					x	x							x	x
Number of 24-hour periods with ≥5 glasses or drinks with alcohol last year (≥12 times/year)	x											x		
**Alcohol problems**														
Have you had periods in life when you drank too much, or at least a bit too much? (3 cat: no, in doubt, yes)		x												
Have you, in ≥1 periods the last 5 years, consumed so much alcohol that it inhibited your work, social life or both? (yes)												x		

Abbreviations: CONOR, Cohort of Norway; HUNT, the Trøndelag Health Study (4S refers to the HUNT4 in South-Trøndelag); NCS, the Norwegian Counties Study; A40P, the Age 40 Program.

[a] Translated from Norwegian to English

*The questionnaire in the Age 40 Program Oslo did not include items covering alcohol consumption

**The questionnaire used in the first two rounds of data collection in the Norwegian Counties Study (1974-1983) and in the Age 40 Program (1985-1994) did not include items covering alcohol consumption

Supplementary Table S5. Proportion of missing information in the included health studies by health study participation[a]

	The Norwegian Counties Study	The Age 40 Program Oslo	The Age 40 Program	The HUNT Study	The Tromsø Study	CONOR
**Number of participants**						
Participation 1	93,568	23,884	416,935	225,914	36,648	80,507
Participation 2	77,853		9,628	68,922	19,909	90
Participation 3	51,620		2,197	38,949	9,647	
Participation 4	18,768		17	19,290	2,812	
**Characteristics**						
**Smoking status (daily)**						
Participation 1	4.3	0.1	0.3	22.0	0.2	0.5
Participation 2	6.1		0.0	5.5	0.6	6.7
Participation 3	8.9		0.0	9.0	1.1	
Participation 4	5.5		5.9	7.3	0.1	
**Smoking intensity (cigarettes/day)**						
Participation 1	5.7	2.4	1.1	26.2	2.1	3.3
Participation 2	7.8		0.9	13.1	6.1	8.9
Participation 3	10.6		0.4	19.7	8.7	
Participation 4	12.3		5.9	20.5	8.8	
**Smoking duration (years)**						
Participation 1	4.5	0.0	0.7	23.8	1.6	2.9
Participation 2	6.6		0.4	8.9	4.8	10.0
Participation 3	9.4		0.7	14.3	6.8	
Participation 4	8.2		5.9	7.8	7.8	
**Leisure-time physical activity**						
Participation 1	4.6	0.7	0.1	11.2	0.2	2.1
Participation 2	6.2		0.0	10.2	2.4	6.7
Participation 3	8.6		0.0	2.5	2.8	
Participation 4	2.3		0.0	6.1	1.1	
**Harmful use of alcohol**						
Participation 1	100.0	100.0	100.0	23.1	18.2	47.5
Participation 2	100.0		100.0	70.5	7.4	44.4
Participation 3	100.0		100.0	5.7	8.2	
Participation 4	100.0		100.0	7.0	7.9	
**Mean alcohol consumption (grams/14 days)**						
Participation 1	100.0	100.0	70.0	40.2	12.7	70.6
Participation 2	100.0		87.0	17.7	15.8	100.0
Participation 3	100.0		21.7	9.9	15.0	

Participation 4	100.0		11.8	17.5	19.2	
**Body mass index**						
Participation 1	8.1	0.2	0.2	6.5	0.2	0.2
Participation 2	9.8		0.2	2.1	0.3	0.0
Participation 3	11.0		0.1	2.1	0.3	
Participation 4	11.1		0.0	5.8	0.6	
**Systolic blood pressure**						
Participation 1	6.6	0.4	0.1	47.3	0.2	0.6
Participation 2	21.6		0.0	1.4	0.2	4.4
Participation 3	22.1		0.1	1.2	0.4	
Participation 4	100.0		0.0	1.2	0.4	
**Diastolic blood pressure**						
Participation 1	6.6	0.5	0.1	47.3	0.2	0.6
Participation 2	21.7		0.0	1.4	0.2	4.4
Participation 3	22.1		0.1	1.3	0.4	
Participation 4	100.0		0.0	1.2	0.4	
**Total cholesterol**						
Participation 1	6.7	0.3	0.1	80.7	0.4	0.2
Participation 2	21.7		0.1	1.4	0.6	0.0
Participation 3	22.2		0.1	2.7	0.8	
Participation 4	100.0		0.0	2.5	0.6	
**Triglycerides**						
Participation 1	6.6	0.5	0.1	80.6	0.4	0.2
Participation 2	21.7		0.1	1.2	0.6	0.0
Participation 3	22.1		0.1	1.8	0.8	
Participation 4	100.0		0.0	2.5	0.6	

Abbreviations: HUNT, The Trøndelag Health Study; CONOR, Cohort of Norway.

[a] In total, 1,197,158 participations. Missing information consists of 77.6% non-measured, non-response (20.7%) and manual cleaning decisions (1.7%)

Supplementary Table S6. Participant characteristics at participation 1, 2, and 3 among individuals with at least 3 participations (n=110,512)[a]

Characteristic	Participation 1	Participation 2	Participation 3
**Age, median (IQR)**	39.4 (11.7)	45.2 (11.4)	53.4 (16.6)
**Sex**			
Woman	51.7	-	-
Man	48.3	-	-
**Birth year, median (IQR)**	1940 (16.7)	-	-
**Participation year, median (IQR)**	1983 (9.5)	1989 (15.6)	1997 (20.8)
**Highest attained education**[b]	109,939	110,022	110,061
Primary	38.3	36.7	35.5
Secondary	46.4	46.0	46.0
Tertiary	15.3	17.4	18.5
**Highest attained income quintile**[b]	103,034	106,130	107,096
Quintile 1	11.4	6.7	5.1
Quintile 2	12.7	9.7	7.4
Quintile 3	20.7	19.1	16.7
Quintile 4	21.4	24.2	24.5
Quintile 5	33.8	40.3	46.3
**Smoking status (daily)**	102,026	106,325	102,502
Never	40.0	39.6	38.9
Former	21.3	26.7	33.3
Current	38.7	33.8	27.7
**Smoking intensity (cigarettes/day)**[c]	98,985	103,083	96,475
Median (IQR)	5.0 (10.0)	6.0 (11.0)	5.0 (12.0)
**Smoking duration (years)**[c]	100,771	104,748	99,528
Median (IQR)	7.0 (18.0)	10.0 (22.0)	12.0 (28.0)
**Leisure-time physical activity**	101,158	105,196	103,929
Category 1	28.2	24.2	23.6
Category 2	51.2	50.4	52.8
Category 3	16.9	21.2	18.1
Category 4	3.7	4.3	5.5
**Harmful use of alcohol**	34,456	17,977	42,712
No	87.7	77.2	85.7
Yes	12.3	22.8	14.3
**Mean alcohol consumption (grams/14 days)**	14,186	37,780	48,725
Median (IQR)	39.1 (68.1)	36.6 (65.6)	38.2 (75.2)
**Body mass index (kg/m^2)**	104,919	105,671	103,947
Median (IQR)	24.2 (4.3)	25.2 (4.8)	26.0 (5.2)
Underweight	1.2	0.7	0.8
Normal weight	58.8	46.5	38.1
Overweight	32.6	40.1	43.1
Obesity	7.4	12.7	18.0
**Blood pressure**	106,062	106,610	98,006
Systolic (mmHg), median (IQR)	128.0 (20.0)	132.0 (23.0)	133.0 (25.0)
Diastolic (mmHg), median (IQR)	80.0 (15.0)	81.0 (15.0)	78.0 (15.0)
**Blood lipids**	71,545	106,297	97,395
Total cholesterol (mmol/L), median (IQR)	5.9 (1.7)	5.9 (1.5)	6.0 (1.6)
Triglycerides (mmol/L), median (IQR)	1.3 (1.0)	1.4 (1.1)	1.5 (1.1)

Abbreviations: IQR, inter-quartile range

[a] Data are presented as column percentages or participants, unless otherwise indicated. Because of rounding, percentages may not sum up to 100%.

[b] At the time of each participation

[c] For current and former smokers

Supplementary Table S7. Participant characteristics by cluster of noncommunicable disease risk factors (n=625,364)[a]

Characteristic	No risk factors	One risk factor	Class 1 *Dyslipidaemia*	Class 2 *Inactive smokers*	Class 3 *Smokers with hypercholesterolaemia*	Class 4 *Obesity*	Class 5 *Hypertension*
**Participants, No. (%)**	236,023 (37.7)	238,986 (38.2)	14,028 (2.2)	45,720 (7.3)	30,954 (4.9)	31,692 (5.1)	27,961 (4.5)
**Sex**							
Woman	55.0	52.4	16.2	54.2	43.4	51.1	39.8
Man	45.0	47.6	83.8	45.8	56.6	48.9	60.2
**Age, median (IQR)**	41.2 (2.2)	41.3 (2.3)	41.4 (2.1)	41.2 (2.1)	41.8 (3.2)	41.5 (2.5)	42.6 (19.4)
**Birth year, median (IQR)**	1951 (9.0)	1951 (9.0)	1951 (8.2)	1951 (8.2)	1947 (13.2)	1953 (9.8)	1944 (22.0)
**Participation year, median (IQR)**	1993 (8.7)	1993 (8.4)	1992 (7.9)	1993 (7.9)	1990 (8.8)	1995 (8.1)	1992 (7.6)
**Highest attained education**[b]	234,453	236,908	13,887	45,226	30,712	31,240	27,726
Primary	16.3	27.9	32.5	35.9	40.8	34.8	41.4
Secondary	45.6	48.8	48.9	49.1	45.1	47.1	44.5
Tertiary	38.1	23.3	18.5	15.1	14.0	18.1	14.1
**Highest attained income quintile**[b]	228,931	231,205	13,656	44,400	29,695	30,375	26,368
Quintile 1	4.0	4.2	3.3	4.5	5.6	5.9	7.4
Quintile 2	5.8	6.8	7.0	7.9	7.9	8.1	8.2
Quintile 3	14.0	16.5	18.2	17.3	17.5	17.1	17.7
Quintile 4	25.3	26.5	26.4	28.0	25.0	26.3	23.0
Quintile 5	50.8	45.9	45.1	42.3	44.0	42.7	43.8
**Smoking status (daily)**	236,023	238,986	14,028	45,720	30,954	31,692	27,961
Never	62.7	27.4	19.0	0.0	10.5	29.2	30.5
Former	37.3	18.2	17.3	0.0	7.7	20.5	21.7
Current	0.0	54.4	63.8	100.0	81.9	50.3	47.8
**Smoking intensity (cigarettes/day)**[c]	233,640	235,795	13,788	45,197	30,508	31,195	27,386
Median (IQR)	0.0 (8.0)	10.0 (15.0)	12.0 (15.0)	15.0 (10.0)	12.0 (9.0)	10.0 (15.0)	10.0 (15.0)
**Smoking duration (years)**[c]	234,351	237,498	13,948	45,453	30,823	31,348	27,746
Median (IQR)	0.0 (8.0)	15.0 (22.0)	20.0 (17.0)	22.0 (7.0)	20.0 (10.0)	15.0 (23.0)	17.0 (25.0)
**Leisure-time physical activity**	236,023	238,986	14,028	45,720	30,954	31,692	27,961
Category 1	0.0	22.4	38.0	100.0	41.4	54.0	37.6
Category 2	64.1	55.2	44.2	0.0	43.9	34.6	47.1
Category 3	28.2	18.9	15.5	0.0	13.0	9.4	13.3
Category 4	7.7	3.5	2.3	0.0	1.6	2.0	2.0
**Harmful use of alcohol**	33,275	27,324	1,110	4,725	2,915	5,712	3,880
No	79.6	78.1	71.0	69.4	79.3	78.0	84.2
Yes	20.4	21.9	29.0	30.6	20.7	22.0	15.8
**Mean alcohol consumption (grams/14 days)**	77,531	73,478	4,242	16,772	7,007	12,785	5,744

Median (IQR)	43.2 (74.5)	43.2 (83.3)	59.5 (104.6)	47.6 (98.8)	47.6 (98.8)	28.8 (86.4)	40.0 (102.0)
**Body mass index (kg/m²)**	236,023	238,986	14,028	45,720	30,954	31,692	27,961
Median (IQR)	24.1 (3.8)	24.4 (4.6)	27.0 (3.6)	23.6 (4.1)	24.8 (3.9)	32.1 (3.3)	27.3 (6.2)
Underweight	0.9	1.3	0.1	2.5	1.0	0.0	0.7
Normal weight	61.8	55.5	23.4	64.4	51.9	0.0	28.8
Overweight	37.3	34.9	64.2	33.1	47.1	0.0	40.3
Obesity	0.0	8.3	12.3	0.0	0.0	100.0	30.3
**Blood pressure**	236,023	238,980	14,028	45,720	30,954	31,692	27,952
Systolic (mmHg), median (IQR)	126.0 (18.0)	128.0 (20.0)	134.0 (16.0)	125.0 (18.0)	130.0 (19.0)	133.0 (18.0)	165.0 (14.0)
Diastolic (mmHg), median (IQR)	75.0 (13.0)	77.0 (14.0)	82.0 (12.0)	76.0 (12.0)	80.0 (12.0)	80.0 (13.0)	100.0 (13.0)
**Blood lipids**	236,023	238,986	14,028	45,720	30,954	31,692	27,961
Total cholesterol (mmol/L), median (IQR)	5.3 (1.2)	5.6 (1.4)	7.1 (1.6)	5.5 (1.2)	7.5 (0.8)	6.0 (1.6)	6.5 (1.8)
Triglycerides (mmol/L), median (IQR)	1.2 (0.8)	1.4 (1.0)	4.9 (1.6)	1.3 (0.9)	1.8 (1.2)	2.1 (1.7)	2.0 (1.6)

Abbreviations: No., number; IQR, inter-quartile range

[a] Data are presented as number of participants or column percentages, unless otherwise indicated. Because of rounding, percentages may not sum up to 100%.

[b] At first participation

[c] For current and former smokers

Supplementary Table S8. Participant characteristics at trajectory entry by latent class of smoking intensity trajectory (n=22,412)[a]

Characteristic[b]	Non-smokers[c]	Class 1 *Medium-to-low*	Class 2 *High-to-low*	Class 3 *Stable high*
**Participants, No. (%)**	12,708 (56.7)	4,386 (19.6)	2,813 (12.6)	2,505 (11.2)
**Sex**				
Woman	53.1	53.3	43.2	50.8
Man	46.9	46.7	56.8	49.2
**Age, median (IQR)**	36.3 (4.0)	35.9 (4.4)	35.9 (4.0)	36.3 (3.7)
**Birth year, median (IQR)**	1945 (10.8)	1945 (11.0)	1945 (10.5)	1942 (8.0)
**Participation year, median (IQR)**	1980 (8.7)	1978 (8.8)	1977 (8.7)	1977 (8.9)
**Highest attained education[d]**	12,662	4,369	2,802	2,493
Primary	23.9	32.6	37.6	42.6
Secondary	52.6	53.0	52.5	50.3
Tertiary	23.5	14.4	9.9	7.1
**Highest attained income quintile[d]**	11,941	4,157	2,719	2,385
Quintile 1	8.4	8.1	6.2	9.1
Quintile 2	9.7	9.9	10.4	11.1
Quintile 3	20.5	23.7	21.2	24.1
Quintile 4	23.2	25.2	27.7	23.9
Quintile 5	38.2	33.1	34.5	31.9
**Smoking status (daily)**	11,627	4,091	2,494	2,247
Never	68.7	4.3	0.1	0.7
Former	30.9	22.2	3.7	4.7
Current	0.5	73.5	96.2	94.6
**Smoking intensity (cigarettes/day)[e]**	11,238	4,016	2,451	2,213
Median (IQR)	0.0 (5.0)	8.0 (5.0)	15.0 (8.0)	12.0 (5.0)
**Smoking duration (years)[e]**	11,415	4,039	2,484	2,239
Median (IQR)	0.0 (4.0)	15.0 (8.0)	17.0 (5.0)	17.0 (6.0)
**Leisure-time physical activity**	11,550	4,075	2,469	2,232
Category 1	25.1	30.2	37.4	32.7
Category 2	53.2	51.1	47.4	50.8
Category 3	18.0	16.6	13.4	15.0
Category 4	3.7	2.1	1.7	1.5
**Harmful use of alcohol**	4,038	1,521	879	635
No	92.5	88.1	82.5	87.2
Yes	7.5	11.9	17.5	12.8
**Mean alcohol consumption (grams/14 days)**	899	266	121	91
Median (IQR)	28.8 (63.8)	39.1 (62.8)	28.8 (71.4)	43.2 (94.2)
**Body mass index (kg/m^2)**	12,174	4,243	2,671	2,377
Median (IQR)	23.8 (3.8)	23.6 (4.0)	23.6 (4.0)	23.5 (3.8)
Underweight	0.8	1.6	1.6	1.8
Normal weight	64.5	66.2	66.7	67.4
Overweight	29.8	27.5	27.6	25.9
Obesity	4.8	4.8	4.1	5.0
**Blood pressure**	12,310	4,285	2,690	2,401
Systolic (mmHg), median (IQR)	126.0 (18.0)	124.0 (18.0)	126.0 (16.0)	124.0 (18.0)
Diastolic (mmHg), median (IQR)	80.0 (12.0)	78.0 (13.0)	80.0 (14.0)	78.0 (14.0)
**Blood lipids**	7,262	2,548	1,586	1,585
Total cholesterol (mmol/L), median (IQR)	5.7 (1.4)	5.8 (1.5)	6.0 (1.5)	6.0 (1.5)
Triglycerides (mmol/L), median (IQR)	1.2 (0.9)	1.3 (1.0)	1.4 (1.1)	1.4 (1.0)

Abbreviations: No., number; IQR, inter-quartile range

[a] Data are presented as number of participants or column percentages, unless otherwise indicated. Because of rounding, percentages may not sum up to 100%.

[b] Participant characteristics are described at trajectory entry, ie the first participation in the period 30-40 years of age.

[c] Never currently daily smokers. A small number of participants (0.5%) reported being current smokers at trajectory entry, but never reported any smoking intensity.

[d] At trajectory entry

[e] For current and former smokers

Supplementary Table S9. Participant characteristics at trajectory entry by latent class of leisure-time physical activity trajectory (n=22,425)[a]

Characteristic[b]	Class 1 *Stable moderate*	Class 2 *Moderate-to-vigorous-to-moderate*	Class 3 *Moderate-to-light-to-moderate*
**Participants, No. (%)**	16,132 (71.9)	2,412 (10.8)	3,881 (17.3)
**Sex**			
Woman	53.6	33.2	54.9
Man	46.4	66.8	45.1
**Age, median (IQR)**	36.2 (3.9)	35.9 (4.8)	36.2 (4.5)
**Birth year, median (IQR)**	1943 (10.0)	1947 (12.0)	1946 (10.0)
**Participation year, median (IQR)**	1977 (8.7)	1984 (8.7)	1984 (8.3)
**Highest attained education**[c]	16,076	2,407	3,855
Primary	30.2	18.9	32.5
Secondary	51.8	52.8	55.0
Tertiary	18.0	28.3	12.6
**Highest attained income quintile**[c]	15,164	2,343	3,707
Quintile 1	8.9	3.4	8.2
Quintile 2	10.1	7.2	11.1
Quintile 3	21.6	20.2	22.8
Quintile 4	23.1	28.2	26.3
Quintile 5	36.3	41.1	31.6
**Smoking status (daily)**	14,796	2,207	3,463
Never	40.0	48.3	34.3
Former	23.0	27.6	19.8
Current	37.0	24.1	46.0
**Smoking intensity (cigarettes/day)**[d]	14,424	2,146	3,348
Median (IQR)	5.0 (10.0)	2.0 (10.0)	8.0 (15.0)
**Smoking duration (years)**[d]	14,619	2,171	3,394
Median (IQR)	7.0 (15.0)	2.0 (13.0)	10.0 (17.0)
**Leisure-time physical activity**	14,708	2,201	3,428
Category 1	21.5	3.5	74.2
Category 2	61.2	31.6	24.9
Category 3	16.2	45.9	0.9
Category 4	1.1	19.0	0.1
**Harmful use of alcohol**	4,421	890	1,765
No	89.8	91.5	89.1
Yes	10.2	8.5	10.9
**Mean alcohol consumption (grams/14 days)**	929	238	212
Median (IQR)	38.2 (66.3)	36.6 (60.1)	25.6 (64.9)
**Body mass index (kg/m^2)**	15,406	2,340	3,732
Median (IQR)	23.7 (3.9)	23.7 (3.2)	24.0 (4.5)
Underweight	1.2	0.6	1.6
Normal weight	65.8	70.4	60.9
Overweight	28.5	27.0	30.3
Obesity	4.5	2.1	7.3
**Blood pressure**	15,568	2,359	3,771
Systolic (mmHg), median (IQR)	126.0 (18.0)	126.0 (18.0)	124.0 (18.0)
Diastolic (mmHg), median (IQR)	80.0 (14.0)	80.0 (14.0)	80.0 (12.0)
**Blood lipids**	10,133	1,291	1,563
Total cholesterol (mmol/L), median (IQR)	5.8 (1.4)	5.6 (1.4)	5.9 (1.5)
Triglycerides (mmol/L), median (IQR)	1.3 (1.0)	1.3 (0.9)	1.3 (1.0)

Abbreviations: No., number; IQR, inter-quartile range

[a] Data are presented as number of participants or column percentages, unless otherwise indicated. Because of rounding, percentages may not sum up to 100%.

[b] Participant characteristics are described at trajectory entry, ie the first participation in the period 30-40 years of age.

[c] At trajectory entry

[d] For current and former smoker

Supplementary Table S10. Participant characteristics at trajectory entry by latent class of body mass index trajectory (n=22,391)[a]

Characteristic[b]	Class 1 *Normal weight*	Class 2 *Normal weight-to-overweight*	Class 3 *Overweight-to-obese*
**Participants, No. (%)**	10,024 (44.8)	10,021 (44.8)	2,346 (10.5)
**Sex**			
Woman	60.0	43.1	52.5
Man	40.0	56.9	47.5
**Age, median (IQR)**	36.3 (3.8)	36.1 (4.2)	36.0 (4.6)
**Birth year, median (IQR)**	1943 (10.3)	1945 (10.5)	1946 (11.2)
**Participation year, median (IQR)**	1977 (8.7)	1981 (8.7)	1984 (8.7)
**Highest attained education**[c]	9,996	9,982	2,330
Primary	27.5	29.7	36.4
Secondary	51.1	54.0	51.8
Tertiary	21.4	16.3	11.8
**Highest attained income quintile**[c]	9,453	9,515	2,216
Quintile 1	8.7	7.0	10.6
Quintile 2	9.9	9.7	11.5
Quintile 3	21.5	21.6	22.3
Quintile 4	23.4	25.0	24.9
Quintile 5	36.4	36.7	30.7
**Smoking status (daily)**	9,235	9,105	2,102
Never	40.8	39.3	38.9
Former	20.4	25.2	24.2
Current	38.8	35.5	37.0
**Smoking intensity (cigarettes/day)**[d]	9,022	8,844	2,030
Median (IQR)	5.0 (10.0)	6.0 (12.0)	6.0 (14.0)
**Smoking duration (years)**[d]	9,134	8,962	2,065
Median (IQR)	7.0 (15.0)	8.0 (15.0)	8.0 (15.0)
**Leisure-time physical activity**	9,181	9,054	2,078
Category 1	25.3	29.6	37.2
Category 2	54.5	50.0	48.2
Category 3	17.1	17.6	12.2
Category 4	3.1	2.8	2.5
**Harmful use of alcohol**	2,818	3,398	860
No	90.5	89.8	88.1
Yes	9.5	10.2	11.9
**Mean alcohol consumption (grams/14 days)**	542	632	205
Median (IQR)	28.8 (63.4)	38.4 (61.3)	38.4 (67.0)
**Body mass index (kg/m^2)**	9,607	9,625	2,249
Median (IQR)	21.9 (2.3)	25.0 (2.6)	29.4 (3.8)
Underweight	2.6	0.0	0.0
Normal weight	94.7	50.3	5.1
Overweight	2.7	48.5	54.6
Obesity	0.0	1.2	40.3
**Blood pressure**	9,709	9,703	2,280
Systolic (mmHg), median (IQR)	124.0 (16.0)	126.0 (18.0)	130.0 (20.0)
Diastolic (mmHg), median (IQR)	78.0 (14.0)	80.0 (12.0)	84.0 (12.0)
**Blood lipids**	6,236	5,552	1,191
Total cholesterol (mmol/L), median (IQR)	5.6 (1.4)	5.8 (1.5)	6.1 (1.6)
Triglycerides (mmol/L), median (IQR)	1.1 (0.8)	1.4 (1.1)	1.6 (1.3)

Abbreviations: No., number; IQR, inter-quartile range

[a] Data are presented as number of participants or column percentages, unless otherwise indicated. Because of rounding, percentages may not sum up to 100%.

[b] Participant characteristics are described at trajectory entry, ie the first participation in the period 30-40 years of age.

[c] At trajectory entry

[d] For current and former smokers

Supplementary Table S11. Participant characteristics at trajectory entry by latent class of blood pressure trajectory (n=22,403)[a]

Characteristic[b]	Class 1 *Slightly increasing systolic and slightly decreasing diastolic*	Class 2 *Increasing-to-decreasing blood pressure*	Class 3 *Increasing blood pressure*
**Participants, No. (%)**	12,169 (54.3)	5,279 (23.6)	4,955 (22.1)
**Sex**			
Woman	59.8	35.8	48.6
Man	40.2	64.2	51.4
**Age, median (IQR)**	36.0 (4.4)	36.2 (3.8)	36.5 (3.7)
**Birth year, median (IQR)**	1945 (11.0)	1943 (9.5)	1943 (10.5)
**Participation year, median (IQR)**	1981 (8.6)	1977 (8.6)	1977 (8.9)
**Highest attained education**[c]	12,119	5,261	4,938
Primary	26.7	33.5	31.7
Secondary	53.1	51.5	51.9
Tertiary	20.2	14.9	16.4
**Highest attained income quintile**[c]	11,522	5,017	4,654
Quintile 1	8.0	7.9	9.0
Quintile 2	10.2	9.7	9.8
Quintile 3	21.3	22.8	21.2
Quintile 4	24.3	23.9	24.5
Quintile 5	36.3	35.7	35.5
**Smoking status (daily)**	11,084	4,827	4,539
Never	39.7	41.5	39.1
Former	22.1	24.5	23.3
Current	38.2	34.1	37.6
**Smoking intensity (cigarettes/day)**[d]	10,772	4,710	4,422
Median (IQR)	5.0 (10.0)	5.0 (12.0)	6.0 (10.0)
**Smoking duration (years)**[d]	10,915	4,771	4,483
Median (IQR)	8.0 (15.0)	7.0 (15.0)	8.0 (16.0)
**Leisure-time physical activity**	11,011	4,801	4,509
Category 1	28.7	27.8	28.5
Category 2	52.3	52.1	50.5
Category 3	16.1	17.5	18.0
Category 4	3.0	2.6	2.9
**Harmful use of alcohol**	3,958	1,565	1,553
No	90.4	89.5	89.0
Yes	9.6	10.5	11.0
**Mean alcohol consumption (grams/14 days)**	907	198	274
Median (IQR)	28.8 (63.8)	37.4 (68.0)	47.6 (66.1)
**Body mass index (kg/m^2)**	11,678	5,046	4,756
Median (IQR)	23.2 (3.5)	24.9 (4.3)	23.9 (3.8)
Underweight	1.5	0.5	1.2
Normal weight	72.3	50.6	64.3
Overweight	23.4	39.2	30.4
Obesity	2.8	9.8	4.2
**Blood pressure**	11,792	5,096	4,813
Systolic (mmHg), median (IQR)	120.0 (14.0)	140.0 (16.0)	126.0 (15.0)
Diastolic (mmHg), median (IQR)	76.0 (10.0)	90.0 (10.0)	80.0 (10.0)
**Blood lipids**	6,916	3,160	2,912
Total cholesterol (mmol/L), median (IQR)	5.6 (1.4)	6.0 (1.5)	5.8 (1.5)
Triglycerides (mmol/L), median (IQR)	1.2 (0.8)	1.5 (1.2)	1.3 (1.0)

Abbreviations: No., number; IQR, inter-quartile range

[a] Data are presented as number of participants or column percentages, unless otherwise indicated. Because of rounding, percentages may not sum up to 100%.

[b] Participant characteristics are described at trajectory entry, ie the first participation in the period 30-40 years of age.

[c] At trajectory entry

[d] For current and former smokers

Supplementary Table S12. Participant characteristics at trajectory entry by latent class of blood lipids trajectory (n=22,400)[a]

Characteristic[b]	Class 1 *Slightly increasing blood lipids*	Class 2 *Increasing-to-decreasing blood lipids*
**Participants, No. (%)**	19,397 (86.6)	3,003 (13.4)
**Sex**		
Woman	56.0	23.9
Man	44.0	76.1
**Age, median (IQR)**	36.2 (4.1)	36.0 (4.1)
**Birth year, median (IQR)**	1945 (10.5)	1945 (10.5)
**Participation year, median (IQR)**	1978 (8.7)	1978 (8.7)
**Highest attained education**[c]	19,328	2,987
Primary	28.3	36.3
Secondary	52.7	51.2
Tertiary	19.0	12.5
**Highest attained income quintile**[c]	18,290	2,900
Quintile 1	8.6	5.7
Quintile 2	10.0	9.6
Quintile 3	21.2	24.2
Quintile 4	23.9	26.5
Quintile 5	36.3	34.0
**Smoking status (daily)**	17,730	2,719
Never	41.7	28.9
Former	23.0	22.5
Current	35.4	48.6
**Smoking intensity (cigarettes/day)**[d]	17,264	2,639
Median (IQR)	5.0 (10.0)	10.0 (15.0)
**Smoking duration (years)**[d]	17,479	2,689
Median (IQR)	6.0 (15.0)	12.0 (18.0)
**Leisure-time physical activity**	17,621	2,699
Category 1	27.7	33.4
Category 2	52.6	47.2
Category 3	16.8	17.0
Category 4	3.0	2.4
**Harmful use of alcohol**	6,086	990
No	90.2	87.9
Yes	9.8	12.1
**Mean alcohol consumption (grams/14 days)**	1,213	166
Median (IQR)	35.7 (66.3)	47.6 (70.9)
**Body mass index (kg/m^2)**	18,609	2,869
Median (IQR)	23.5 (3.7)	25.5 (4.1)
Underweight	1.3	0.4
Normal weight	69.0	42.7
Overweight	25.8	47.2
Obesity	4.0	9.8
**Blood pressure**	18,803	2,896
Systolic (mmHg), median (IQR)	124.0 (18.0)	130.0 (18.0)
Diastolic (mmHg), median (IQR)	78.0 (14.0)	84.0 (14.0)
**Blood lipids**	11,297	1,691
Total cholesterol (mmol/L), median (IQR)	5.6 (1.3)	7.2 (1.5)
Triglycerides (mmol/L), median (IQR)	1.2 (0.8)	2.4 (1.8)

Abbreviations: No., number; IQR, inter-quartile range

[a] Data are presented as number of participants or column percentages, unless otherwise indicated. Because of rounding, percentages may not sum up to 100%.

[b] Participant characteristics are described at trajectory entry, i.e. the first participation in the period 30-40 years of age.

[c] At trajectory entry

[d] For current and former smokers
